# New energy vehicles’ technology innovation coordination strategy based on alliance negotiation under dual credit policy

**DOI:** 10.1371/journal.pone.0299915

**Published:** 2024-03-15

**Authors:** Miaomiao Ma, Weidong Meng, Bo Huang, Yuyu Li

**Affiliations:** 1 School of Management, Chongqing Institute of Engineering, Chongqing, China; 2 School of Economics and Business Administration, Chongqing University, Chongqing, China; 3 School of Economics and Management, Chongqing Normal University, Chongqing, China; University of Duhok, IRAQ

## Abstract

The development of new energy vehicles (NEVs) is one of the effective ways to alleviate carbon emissions, environmental pollution, and energy scarcity in the transportation sector. The Chinese government has innovatively proposed the “dual credit policy,” but it is still a hot topic whether it can promote the NEVs’ technological innovation. In this study, we construct game models and obtain the technological innovation strategies for NEVs under the dual credit policy, considering that the NEV supply chain contains one manufacturer and N suppliers. Further, we construct bargaining game models and study how to encourage manufacturers and suppliers to enhance technological innovation, realize supply chain coordination, and give the alliance strategy to maximize suppliers’ profit. We found that the dual credit policy can effectively stimulate technological innovation, and the higher the credit price or technological innovation credit factor, the higher the technical level of NEVs. The findings could guide the government to adjust and revise the policy. Second, we found that the bargaining games could coordinate the NEV supply chain so that decentralized enterprises can achieve optimal technological innovation under centralized decision-making. Third, we found that suppliers can improve their profits by choosing a suitable alliance strategy under the manufacturer’s different negotiating power.

## 1. Introduction

Various countries have vigorously promoted new energy vehicles (NEVs) due to their remarkable advantages of low-carbon emissions, environmental protection, and energy-saving [1]. In China, NEVs are developing rapidly under the fiscal subsidy policy [2]. However, as the consumption of NEVs continues to grow, the financial subsidy of NEVs has brought a heavy burden to the Chinese government [3]. In addition, manufacturers focus on obtaining short-term benefits through subsidy policies but poorly focus on improving the technical performance of NEVs, resulting in a sharp decrease in the fiscal subsidies’ efficiency [4]. In 2017, the Ministry of Industry and Information Technology and five other ministries and commissions jointly proposed the “Parallel Management Measures for the Average Fuel Consumption of Passenger Vehicle Enterprises and Credits for New Energy Vehicles” (referred “dual credit policy”) [5].

Under the dual credit policy, each NEV could obtain a certain number of credits, and the manufacturer obtains revenue by selling the credits. The higher the NEVs’ technology performance is, the more credits they have and the higher the credit revenue. The dual credit policy aims to promote the competitiveness of the core products and key components by driving the market force and guiding the industry’s sustainable growth. However, whether the dual credit policy can achieve the desired effect is still debated.

The NEVs’ technology performance improvement depends on these components’ technology improvement, breakthroughs, and suppliers’ innovation investment. However, automobile manufacturers enjoy credit income from technological innovation under the policy. The suppliers of the parts bear the investment costs of technological innovation, which leads to a lack of willingness and enthusiasm from suppliers to engage in technological innovation. Therefore, it is the key to encouraging suppliers to increase technological innovation.

This paper makes three main contributions. Firstly, under the dual credit policy, we established a game-theoretic model for technological innovation in the NEVs supply chain. Through rigorous theoretical derivation, we demonstrated the incentive role of the dual credit policy in stimulating NEVs’ technological innovation, providing a theoretical basis for the government to revise the dual credit policy. Secondly, by designing a profit-sharing mechanism based on supplier alliances, it was possible to effectively incentivize suppliers to increase their investment in NEVs’ technological innovation, optimizing the incentive effects of the dual credit policy. Thirdly, we demonstrated that suppliers can choose different alliance strategies under different negotiating powers to maximize their profits, providing theoretical guidance for supplier alliance decision-making.

## 2. Literature review

This research involves three streams, the NEV supply chain, dual credit policy, and supply chain coordination.

### 2.1 The NEV supply chain

With the development of NEVs, scholars have conducted extensive research on the production and R&D strategies for the NEVs supply chain.

Some scholars pay attention to the production strategies of the NEVs supply chain in different situations. For example, Liu and Wang studied the optimal decision-making of the NEVs closed-loop supply chain under different government subsidy policies, considering that battery suppliers occupy power battery recycling channels [6]. Gu et al. studied the optimal pricing strategies of manufacturers and remanufacturers by considering the NEVs closed-loop supply chain including the recycling and reuse of electric vehicle batteries [7]. Zhao et al. studied the pricing strategy of the NEVs supply chain under decentralized and centralized decision-making, and further studied the impact of charging facilities on the NEVs’ pricing [8]. Ma et al. studied the impact of cost sharing contract and responsibility sharing contract on the sales price of NEVs, and found that the dual-channel recycling mode can always reduce the NEVs’ market price [9]. Gong et al. studied the impact of consumers’ low-carbon preference on the pricing decision of the NEVs supply chain and found that the higher the proportion of consumers’ low-carbon preference and green consumers, the higher the NEVs’ price [10]. Wu et al. considered government subsidies and different market power structures and found that the increase in recycling price may lead to an increase in the NEVs’ sales price [11]. Wang et al. studied the selection of cooperative competition modes in the NEVs supply chain and found that cooperative competition for patent licensing was not necessarily beneficial to supply chain members [12].

Some scholars pay attention to the R&D strategies of the NEVs supply chain in different situations. For example, Chen believed that the application of digital twin technology to strengthen investment in general technology could reduce the R&D cost of NEVs, thus promoting the development of the NEV industry [13]. Zhu et al. considered the existence of competition among manufacturers and discovered that competition among manufacturers may reduce the technology R&D of upstream battery suppliers [14]. Zhu et al. compared the impact of government subsidies and carbon trading policies on R&D decision-making in the NEVs supply chain, and found that only when subsidies are based on actual driving range, government subsidies can increase technology R&D investment. Otherwise, the incentive effect of carbon trading policy is better [15]. Sun et al. studied the optimal technology R&D and advertising investment in the NEVs supply chain, considering the influence of government subsidies and advertising [16]. Zhu et al. studied the impact of equity ownership on the R&D and innovation of NEVs, and found that battery suppliers would benefit from cross-shareholding in the supply chain, and cross-shareholding provided the greatest incentive for R&D of NEV batteries [17]. Liu et al. studied the impact of different shareholding strategies on the vertical cooperative innovation of the NEVs supply chain, and found that vertical shareholding is conducive to improving the battery endurance, but battery suppliers are more inclined to choose cross-shareholding strategies [18]. Shi and Hu studied the impact of differentiated government subsidies and information asymmetry on the NEVs supply chain and found that differentiated subsidies can always encourage manufacturers to improve the technical level of NEVs [19].

### 2.2 Dual credit policy

Since the dual credit policy’s implementation, there have been a few studies on the effect of dual credit policy, but the research conclusions are inconsistent. Some scholars believe that the dual credit policy has positively promoted the technological innovation of NEVs. Such as, Li et al. believed that the dual credit policy was more effective than the subsidy policy considering the battery recycling of NEVs [20]. Ma et al. showed that implementing the policy has significantly increased the R&D investment and improved the technical level of NEVs [21]. Dong and Zheng believed that the policy could significantly improve the technological productivity of enterprises [22]. Wang et al. found that the policy can enhance the enterprises’ willingness to carry out green technology innovation, and increasing the credit price can make enterprises’ economic and environmental interests consistent [23]. Yang et al. believed that the policy positively promotes the development of NEVs, and maintaining a moderate credit price can improve the NEVs’ technical level [24]. Zhou et al. found that the policy increased the green technology threshold of NEVs [25]. Yang et al. showed that the policy is conducive to energy conservation and emission reduction in transportation and promotes the development of NEVs [26].

Some scholars believe that the dual credit policy has not yet achieved the expected effect, and further adjustments and revisions are needed. For instance, Ou et al. concluded that a separate CAFC policy promotes PEV sales more than the dual credit policy [27]. Lou et al. believed that the dual credit policy cannot improve the fuel economy of fuel cars and is not conducive to producing fuel-efficient vehicles. The government should set different NEV standards for different automobile manufacturers [28]. Cheng and Fan believed higher credit prices promote the NEVs’ development more than setting higher output rates under a dual credit policy [29]. He et al. showed that setting the upper and lower limits of the credit price or gradually tightening the CAFC rules by adjusting the dual credit policy will effectively promote the R&D intensity of electric vehicles [30]. Kong et al. found that the dual credit policy can promote the sustainable development of the automobile industry, but the incentive effect on NEVs is limited [31]. Zhao et al. believed that the policy could promote the NEVs’ technological innovation, but this policy may lose its role in promoting technological innovation in 2025 or earlier [32]. Meng et al. argued that the policy could not promote NEVs’ technology innovation when R&D capital constrained the suppliers’ R&D investment [33]. Li et al. found that due to the current low credit standard and credit price, it is difficult for a dual credit policy to play an incentive role alone, and a compound mechanism of multiple policies, such as subsidies and tax incentives, is required to achieve more substantial incentive effects. Through the literature review, we find that scholars still have certain disputes over the impact of the policy on NEVs [34].

### 2.3 The supply chain coordination

Coordination between manufacturers and suppliers is critical in upstream and downstream enterprises. Many scholars have designed a series of contracts to study the coordination of a supply chain. Such as, Li et al. introduced sales rebate contracts to study the emission reduction cooperation mechanism of low-carbon supply chains and explore the impact of contracts on supply chain performance [35]. He et al., studied an online shopping supply chain composed of a single online retailer and a third-party logistics enterprise and designed bilateral effort cost-sharing contracts to realize the coordination of the supply chain [36]. Wang et al. considered consumers’ low carbon preferences and studied the supply chain coordination under wholesale price and cost-sharing contracts [37]. Cachon and Lariviere argued that revenue-sharing contracts could coordinate a supply chain with a single retailer and arbitrarily distribute the supply chain’s profits [38]. He et al. studied the coordination of various contract types, such as supplier-managed inventory partnership and production subsidy contracts, revenue-sharing contracts with early purchase discounts, return policy and wholesale price contracts, return policy, and sales discount and penalty contracts, in a supply chain when supply and demand are uncertain [39].

The Nash bargaining model is the most popular model used to analyze cooperation [40]. Scholars have studied cooperation in the supply chain environment based on the bargaining game model. For example, Gou and Iyer believed that when the sales price difference of the retailer was minimal, the manufacturer would choose to negotiate on the spot; however, when the price difference was substantial, the manufacturer would choose to negotiate in progression [41]. Feng and Lu analyzed a supply chain’s outsourcing decision and contract choice based on the bargaining model [42, 43]. Basak compared and analyzed a competitive model in which competitive enterprises can obtain optimal profits based on the bargaining model [44]. Zhang et al. researched the influence of enterprises’ bargaining power and centralized procurement efficiency on their willingness to participate in centralized procurement in China’s pharmaceutical market [45]. Escapa and Gutierrez quantitatively studied the distribution of the potential benefits of environmental cooperation between countries based on the bargaining model [46]. Unlike the above studies, this paper studies the coordination of the NEVs supply chain based on the bargaining model under the dual credit policy. It discusses the impact of the negotiating power, negotiation sequence, negotiation sequence decision, and supplier alliance strategy on the NEVs supply chain.

From the literature review, first, scholars have conducted extensive studies on the production and R&D decision-making of the NEV supply chain, but these studies have ignored the impact of the dual credit policy. Dual credit policy directly encourages manufacturers’ technological innovation, and suppliers in the NEV supply chain bear high technological innovation costs. How will implementing a dual credit policy affect relevant decisions in the NEV supply chain? However, there are few relevant studies, and the conclusions are inconsistent. Therefore, what is the incentive effect of implementing the dual credit policy on the technological innovation of NEVs, and how do we achieve the optimal incentive effect of the policy? These are issues that need further discussion. Secondly, scholars have studied the supply chain’s coordination mechanism from different perspectives, providing a solid theoretical and methodological reference for analyzing the coordination of the NEV supply chain. However, existing research mainly focuses on enterprises’ one-on-one cooperation and competition, lacking consideration of reality. In the NEV supply chain, manufacturers usually cooperate with many suppliers. There are complex competitive and cooperative relationships between multiple suppliers and manufacturers. How manufacturers and suppliers cooperate in technological innovation and achieve supply chain coordination is also an urgent issue to be studied. This paper designs centralized decision-making and profit-sharing mechanisms, which effectively motivate suppliers to increase investment in NEVs’ technological innovation, which provides a theoretical frame for future research.

## 3. Problem description and model

### 3.1 Problem description

This study considers that the NEVs supply chain contains one manufacturer and N parts suppliers. Considering N suppliers supplying N parts to one manufacturer, the manufacturer buys parts from suppliers for production and sells NEVs to customers in the final market.

There are two modes that manufacturers purchase parts from suppliers. One is the traditional parts procurement mode. That is, according to the production needs of NEVs, manufacturers purchase parts from each parts manufacturer for production. Another model is modular procurement; suppliers cooperate to produce prefabricated parts modules, and manufacturers purchase parts modules for assembly production. For example, Mercedes-Benz/Swatch uses approximately 25 module suppliers when designing and producing its smart car, while a typical carmaker might use 200–300 suppliers. Nissan uses several supplier alliances to supply parts for its truck plant in Mississippi.

When manufacturers adopt a modular procurement approach, suppliers cooperate in advance to produce parts modules. Since suppliers are the only parts suppliers, there is no competition and substitution relationship between suppliers. In addition, from the perspective of producing parts modules, the lack of any part could not work properly, parts suppliers are equally important.

Under the dual credit policy, manufacturers’ and suppliers’ technological innovation will affect the NEV credits, and they can choose decentralized or centralized decision-making. Under decentralized decision-making, the manufacturer and supplier make decisions on the technological innovation investment and price through the Stackelberg game model to maximize their profits. Under centralized decision-making, enterprises aim to maximize the total profits, jointly determine technological innovation investment, and then determine the parts prices and profit distribution through a bargaining game.

The decentralized decision-making process contains two stages. First, the manufacturer determines its technological innovation and NEVs’ price to maximize its profit. Second, suppliers simultaneously determine their technological innovation and the components’ prices and sign cooperation contracts with manufacturers.

The centralized decision-making process contains three stages. First, suppliers decide the alliance strategy and the profit allocation rules within the alliance. Second, the manufacturer and suppliers jointly make decisions on technological innovation, the sales price, and the negotiation order of NEVs to maximize the total profit of the NEVs supply chain. Since all suppliers (alliances) need to bargain with the manufacturer, the manufacturer has the right to decide on the negotiation order. The manufacturer will negotiate with suppliers individually if the suppliers form multiple alliances. The suppliers then allocate profits within the supplier alliance according to agreed rules, determining each supplier’s profit. If suppliers form a grand alliance, the manufacturer first negotiates with the alliance to determine the manufacturer’s and the alliance’s profits and then distribute the profits within the supplier alliance according to agreed rules. Finally, according to the contract, the NEVs supply chain makes technological innovations, purchases, production, and sales to realize their respective profits.

### 3.2 Model

The NEVs’ market demand is $q=a-p+\theta (T_{m}+\sum_{j=1}^{n}T_{j}$) [47, 48], where *a* is the market demand, *p* is the NEVs price, and *θ* is the consumers’ preference. The more significant *θ* indicates the greater the impact of technical performance on market demand. The subscript *j* indicates the supplier *j* = 1,2⋯,*n*, and the subscript *m* indicates the manufacturer.

The NEVs credits can trade under the dual credit policy, and the credit revenues influence the manufacturer’s profits. According to the dual credit policy, every NEVs can get NEVs credit *σ* on the technical performance *T*_0_. Furthermore, the manufacturer and suppliers conduct technological innovation $T_{m}+\sum_{j=1}^{n}T_{j}$ to prompt the NEVs’ technical performance and the NEVs credit increases $\lambda (T_{m}+\sum_{j=1}^{n}T_{j})$, where *λ* is the credit factor of technological innovation, and *λ* > 0. Therefore, the NEVs credit obtained is $\sigma +\lambda (T_{m}+\sum_{j=1}^{n}T_{j})$. For simplicity of calculation, let *σ* = 0. Set *p*_*e*_ as the credits price, the NEVs credit revenue is $\lambda p_{e}(T_{m}+\sum_{j=1}^{n}T_{j})q$.

The components suppliers’ technological innovation investments are $\frac{1}{2}k_{j}T_{j}^{2}$, where *k*_*j*_>0 (*j* = 1,2,⋯*n*), and *k*_*j*_ is the suppliers’ technological innovation factor. The manufacturer’s technological innovation investment is $\frac{1}{2}k_{m}T_{m}^{2}$, where *k*_*m*_>0, and *k*_*m*_ is the manufacturer’s technological innovation investment factor. The production costs for the manufacturer and supplier are set to 0.

Research shows that since the supplies’ technical innovation investment on NEVs, is a one-time investment, it is far larger than other parameters [49]. To ensure an optimal solution, we assume that $\prod_{j=1}^{n}k_{j}-\sum_{\delta =1}^{n}\frac{\prod_{j=1}^{n}k_{j}}{k_{\delta }}k_{m}{\left(\theta +p_{e}\lambda \right)}^{2}>0$.

So, the supplier’s profit is

$$\pi_{j}=w_{j}q-\frac{1}{2}k_{j}T_{j}^{2}\;j=1,2,\cdots ,n$$ (1)

The manufacturer’s profits consist of the NEVs sales revenue, credit revenues, and technological innovation investment.

The manufacturer’s profit function is

$$\pi_{m}=(p-\sum_{j=1}^{n}w_{j})q+\lambda p_{e}(T_{m}+\sum_{j=1}^{n}T_{j})q-\frac{1}{2}k_{m}T_{m}^{2}$$ (2)

where $p=\omega +\sum_{j=1}^{n}w_{j}\;w_{j}$ is the supliers’ parts price, and *ω* is the manufacturer’s profits markup.

## 4. Model analysis

### 4.1 Optimal solution of decentralized decision

Under decentralized decision-making (marked with D), manufacturers and suppliers decide on technological innovation through the Stackelberg game to maximize their profits.

This section obtains the optimal solution by using the reverse induction method.

First, the manufacturer decides on technological innovation and the NEVs’ price to maximize profits. Second, the suppliers determine their technological innovation and part prices to maximize profits.

The optimal strategy-solving process under decentralized decisions is detailed in Appendix A in S1 File, and the optimal strategy is $T_{j}^{D*}=\frac{a\theta Ζk_{m}}{k_{j}(Ζ(2nk_{m}+Ν)-2\theta (\theta +p_{e}\lambda )M)},\;\pi_{m}^{D*}=\frac{a^{2}k_{m}Ζ}{2(Ζ(2nk_{m}+Ν)-2\theta (\theta +p_{e}\lambda )M)},\;\pi_{j}^{D*}=\frac{a^{2}k_{m}^{2}{Ζ}^{2}(2k_{j}-\theta^{2})}{2k_{j}{\left(Ζ(2nk_{m}+Ν)-2\theta (\theta +p_{e}\lambda )M\right)}^{2}},\;\pi^{D*}=\frac{a^{2}k_{m}Ζ(Ζ(4nk_{m}+Ν)-\theta (3\theta +2p_{e}\lambda )M)}{2{\left(Ζ(2nk_{m}+Ν)-2\theta (\theta +p_{e}\lambda )M\right)}^{2}}$.

### 4.2 Optimal solution of centralized decision

Under centralized decision-making (marked by C), all firms jointly determine the technological innovation to maximize the profit of the NEVs supply chain.

The supply chain’s profit function is

$$\pi^{C}=pq+p_{e}\lambda (\sum_{j=1}^{n}T_{j}+T_{m})q-\frac{1}{2}\sum_{j=1}^{n}k_{j}T_{j}^{2}-\frac{1}{2}k_{m}T_{m}^{2}$$ (3)

Under centralized decision-making, all firms jointly determine the technological innovation and NEVs’ price to maximize the profits of the entire NEVs supply chain.

The optimal strategy-solving process under centralized decisions is detailed in Appendix B in S1 File, and the optimal strategy is $\pi^{C*}=\frac{a^{2}k_{m}Ζ}{2(ΖΝ-{\left(\theta +p_{e}\lambda \right)}^{2}M)},\;q^{C*}=\frac{aΖk_{m}}{ΖΝ-{\left(\theta +p_{e}\lambda \right)}^{2}M},\;T_{j}^{C*}=\frac{{ak}_{m}Ζ(\theta +p_{e}\lambda )}{k_{i}(ΖΝ-{\left(\theta +p_{e}\lambda \right)}^{2}M)},\;T_{m}^{C*}=\frac{aΖ(\theta +p_{e}\lambda )}{ΖΝ-{\left(\theta +p_{e}\lambda \right)}^{2}M}$.

### 4.3 Optimal solution analysis

**Proposition 1.** Under the dual credit policy, the increase of *p*_*e*_ or *λ*, the technological innovation, the profits, the technical performance, and production all increase, regardless of the decision mode.

For proof of Proposition 1, see Appendix C in S1 File.

The policy influences the optimal technological innovation strategy for the NEVs supply chain. With the increase of the credit factor of technological innovation or the credits price, technological innovation, profits, technical performance, and production increase. Therefore, the government must set a reasonable credit price or the credit factor for technological innovation to develop the NEVs industry.

**Proposition 2.** *Compared with the decentralized decision*, *the profits*, *the technological innovation*, *the technical performance*, *and the production of the NEVs are all improved in the centralized decision*. *Under the dual credit policy*, *the advantages of centralized decision-making are more pronounced as the credit price or the credit factor of technological innovation increases*.

For proof of Proposition 2, see Appendix D in S1 File.

Compared with centralized decision-making, decentralized decision-making leads to dysfunctional decision-making and profit loss in the NEVs supply chain due to the dual marginal effect, which decreases the technological innovation of manufacturers and suppliers, and the profit of the NEVs supply chain decreases. In addition, the government can promote the NEVs industry’s sustainable development by adjusting the credit factor of technological innovation or the credit price and guiding the centralized decision-making in the NEVs supply chain.

## 5. The coordination strategy for NEVs supply chain

Designing a coordination strategy that enables independent firms to achieve an optimal centralized decision-making strategy is necessary.

The profit increment is $\Delta \pi =\frac{a^{2}Ζk_{m}(4nk_{m}Ζ(nZ-\theta^{2}Μ)+{{(p}_{e}\lambda )}^{2}Μ(Ζ(4nk_{m}+Ν)-\theta (3\theta +2p_{e}\lambda )+\theta^{4}{Μ}^{2})}{2{\left(Ζ(2nk_{m}+Ν)-2\theta (\theta +p_{e}\lambda )Μ\right)}^{2}(ΖΝ-{\left(\theta +p_{e}\lambda \right)}^{2}Μ)}$.

Therefore, coordination can be realized when Δ*π* is reasonably allocated which guarantees that the profits are not lower than those under decentralized decisions.

The manufacturer and N suppliers determine the allocation of incremental profits through the negotiation game, indicated by *π*_*m*_ and π_j_, respectively. Clearly, $\pi_{m}+\sum_{j=1}^{n}\pi_{j}=\Delta \pi$. The rules of negotiation are that the manufacturer and the supplier take turns to negotiate until the manufacturer and the last supplier negotiate. After the end of the first round of negotiations, the supplier obtains a certain amount of incremental profits. The manufacturer then splits the remaining profit with the remaining N-1 suppliers, and so on. In addition, according to the literature [50, 51] (Nagarajan and Bossok, 2008; Su and Liu, 2010), each component has the same status in the value composition of the final product. Thus, each supplier has the same bargaining power in negotiations with the manufacturer.

Since the manufacturer negotiates with suppliers one by one, and each negotiation game model is

$$Max{\left(\pi_{m}-d_{m}\right)}^{\alpha_{j}}{\left(\pi_{i}-d_{i}\right)}^{\beta_{j}}\;j=1,2,\cdots ,n$$

$$s.t.\;(\pi_{m},\pi_{j})\geq (d_{m},d_{j})$$

$$\pi_{m}+\pi_{j}\leq \Pi_{i}$$ (4)

where Π_*i*_ is the profits that can be allocated in the i^th^ round of negotiations, $\Pi_{1}=\Delta \pi ,\;\Pi_{i}=\Pi_{i-1}-\pi_{\left(j,i-1\right)}$, and the double subscript (*j*,*i*) indicates the supplier *j* negotiates in *i*^*th*^ round. (*d*_*m*_, *d*_*j*_) are the profits that manufacturer and supplier *j* can get when negotiations break down. For simplicity, assume *d*_*m*_ = *d*_*j*_ = 0 in this study. *α*_*j*_(0<*α*_*j*_<1) indicates the manufacturer’s negotiating power. *β*_*j*_(0<*β*_*j*_<1) indicates the supplier`s negotiating power and meets *α*_*j*_+*β*_*j*_ = 1.

Therefore, in the i^th^ round, the supplier’s profit is *β*_*j*_Π_*i*_, and manufacturer’s profit is *α*_*j*_Π_*i*_.

### 5.1 Supplier bargains with the manufacturer independently

When N suppliers negotiate independently, the manufacturer with the suppliers conducts N rounds of negotiations sequentially. Considering that the suppliers provide complementary parts to the manufacturer and are the only supplier, the importance of parts is generally the same, and the negotiating power of the parts suppliers is less different. Therefore, without any loss of generality, we further assume that suppliers have the same negotiating power *β*_*j*_ = *β*(*j* = 1,2,…,*n*).

**Proposition 3.** *When suppliers bargains with manufacturer independently*, the suppliers’ final profits are $\pi_{j}^{*}=\beta \alpha^{n-1}\Delta \pi +\pi_{j}^{D*}$, and the manufacturer’s final profit is $\pi_{m}^{*}=(1-n\beta \alpha^{n-1})\Delta \pi +\pi_{m}^{D*}$.

*Proof*. When the first supplier participates in the negotiations, the profits available for distribution are Π_1_ = Δ*π*. After the first round, the profits of the supplier, the profits of the manufacturer, and the profits available for distribution in the second round of negotiations are $\beta \Delta \pi +\pi_{j}^{D*},\;\alpha \Delta \pi +\pi_{m}^{D*},\;\alpha \Delta \pi$, respectively. After the second negotiations, the profits of the supplier and profits of the manufacturer, and the profits available for distribution in the third round of negotiation are $\alpha \beta \Delta \pi +\pi_{j}^{D*},\alpha^{2}\Delta \pi +\pi_{m}^{D*}$, and *α*^2^Δ*π*, respectively. By analogy, it can prove that if the supplier participates in the *i*^*th*^ round negotiations, the supplier’s profit is $\beta \alpha^{i-1}\Delta \pi +\pi_{j}^{D*}$, and the manufacturer’s profit is $\alpha^{i}\Delta \pi +\pi_{m}^{D*}$.

Without any loss of generality, suppose that supplier participates in the (i−*ε*)^*th*^ negotiations (*i* = 1,2,⋯,*n*; *j* = 2,3,⋯,*n*; and *ε*∈{1,2,⋯,*i*−1}). The supplier’s profit is $\beta \Pi_{i-\varepsilon }+\pi_{j}^{D*}$, which participates in the (i−*ε*)^*th*^ round of negotiations, and supplier’s profit is $\beta \Pi_{i}+\pi_{j}^{D*}$, which participates in the *i*^*th*^ round of negotiations. Comparing the supplier’s profit in (i−*ε*)^*th*^ round and *i*^*th*^ round, we can obtain that $\Delta \pi_{j}=\beta (\Pi_{i-\varepsilon }-\Pi_{i})=\beta \alpha^{i-\varepsilon -1}(1-\alpha^{\varepsilon })>0$, and the profits of the suppliers participating in the (i−*ε*)^*th*^ round of negotiations is more than that in the *i*^*th*^ round. It shows that the suppliers can obtain more profits by negotiating earlier. Similarly, the delayed negotiation order will lead to suppliers’ profits reduce. As a result, suppliers’ profits are affected by their bargaining power and the order in which they negotiate. However, after the *i*^*th*^ round of negotiations, the manufacturer’s profits are $\alpha^{i}\Delta \pi +\pi_{m}^{D*}$, and only his negotiating power affects the profits.

Denoted the supplier’s strategy as *ST*_*i*_ = (*F*_1_, *F*_2_,⋯,*F*_*i*_). (*i* = 1,2,⋯,*n*), where P_*j*_ is the profit that the supplier needs to give to the manufacturer to negotiate in the *i*^*th*^ round. We know that the suppliers`profits in the *i*^*th*^ round is βα^*i*−1^Δπ. After giving profits to the manufacturer, the final profits the suppliers can obtain are $\beta \alpha^{i-1}\Delta \pi -F_{i}+\pi_{j}^{D*}$. When the supplier’s negotiation round is in the last, the supplier’s profits are $\beta \alpha^{n-1}\Delta \pi +\pi_{j}^{D*}$, which are the lowest profits obtained by supplier negotiations. So, *F*_n_ = 0. The upper bound on the supplier’s transfer profit is the entire excess profit gained by bringing forward the negotiation order to the manufacturer, so the supplier’s transfer profits are ${\beta (\alpha }^{i-1}-\alpha^{n-1})\Delta \pi$.

If the supplier negotiates in the *i*^*th*^ round, its profits are $\beta \alpha^{i-1}\Delta \pi +\pi_{j}^{D*}$. The supplier must transfer the profits $F_{i}^{*}$ to negotiate in the *i*^*th*^ round and his final profits are $\beta \alpha^{i-1}\Delta \pi -F_{i}^{*}+\pi_{j}^{D*}.\;F_{i}^{*}$ is suppliers’ transferred profits. Therefore, the supplier can only obtain the last round of negotiations, which is the lowest profits that the supplier can obtain $\beta \alpha^{n-1}\Delta \pi +\pi_{j}^{D*}$. The manufacturer’s profits are $(1-n\beta \alpha^{n-1})\Delta \pi +\pi_{m}^{D*}$, and it is the total of the negotiated profits and the transferred profits of all suppliers.

Q.E.D.

When the suppliers independently negotiate sequentially, their negotiation power and the negotiation order affect the profits. The stronger the suppliers’ negotiating power, the greater the profit gained in advance of the negotiation sequence. However, the manufacturer has the right to determine the order of negotiation. Therefore, the supplier must transfer profits to the manufacturer to negotiate earlier. The most significant profit transfer is the excess profit that the supplier gains by negotiating earlier. In this case, the supplier only gets the last round of negotiations, the lowest profit the supplier can obtain.

### 5.2 Suppliers form multiple alliances to negotiate with manufactures

The above study finds that suppliers can only get the lowest profits since the suppliers do not have the right to decide on the negotiation order. To increase profits, the farsighted suppliers can form alliances for cooperation. We use a set N to represent all suppliers, denoted as *N* = {1,2,⋯,*n*}, where suppliers freely form supplier alliances $B_{\hbar },\;B_{\hbar }\subset N(j=1,2,\cdots ,\xi ),\;\xi$ is the number of supplier alliances, and the value range of *ξ* is 1≤*ξ*≤*n*. When *ξ* = 1, all suppliers form a grand alliance ℬ* = {1,2,⋯,*n*}; and when *ξ* = *n*, suppliers form independent alliances $\bar{B}=\{\{1\},\{2\},\cdots ,\{n\}\}$. We numbered the supplier alliances according to the negotiation order, and ℬ_ℏ_ is the alliance that participated in the ℏ^*th*^ round of negotiations. η_ℏ_ is the number of suppliers in the alliance ℬ_ℏ_. Clearly, η_1_+η_2_+⋯η_*ξ*_ = *n*. When η_1_ = η_2_ = ⋯ = η_*ξ*_, the number of suppliers in each supplier alliance is equal, and we believe that the number of equal-sized supplier alliances is *ξ*.

When suppliers form multiple alliances to negotiate with the manufacturer, the latter conducts *ξ* rounds of pair-to-pair negotiation games with supplier alliance ℬ_ℏ_(ℏ = 1,2,⋯,*ξ*) sequentially. We further assume that the supplier alliance’s negotiating power is the average of the negotiating power of its members [52], i.e., $\beta_{B_{\hbar }}=\frac{\sum_{j=1}^{\eta_{\hbar }}\beta_{j}}{\eta_{\hbar }}=\beta$, $\alpha_{B_{\hbar }}=\frac{\sum_{j=1}^{\eta_{\hbar }}\alpha_{j}}{\eta_{\hbar }}=\alpha$, where *j* = 1,2,⋯,*η*_*ℏ*_; ℏ = 1,2,⋯,*ξ*; and $\alpha_{B_{\hbar }}+\beta_{B_{\hbar }}=1$.

Consistent with the literature [51], each supplier alliance’s negotiation power is equal; thus, the negotiating power of each member in the alliance is equal, and the suppliers in the alliance distribute the alliance profits equally.

**Proposition 4.** When the suppliers form multiple alliances to negotiate with the manufacturer, the final profits of the suppliers are $\pi_{j}^{**}=\beta \alpha^{\xi -1}/\eta_{\hbar }+\pi_{j}^{D*}$, the final profits of the manufacturer are $\pi_{m}^{**}=(1-\xi \beta \alpha^{\xi -1})\Delta \pi +\pi_{m}^{D*}$.

*Proof*. When supplier alliance ℬ_ℏ_ participates in the first round, the available profits are Π_1_ = Δ*π*. After the first negotiations, the supplier alliance’s profit is ℬ_ℏ_, the profits of the manufacturer and the profits available for distribution in the second round of negotiations are *α*Δ*π*, *β*Δ*π*, and *α*Δ*π*, respectively; and the profits of suppliers are $\beta \Delta \pi /\eta_{\hbar }{+\pi }_{j}^{D*}$. After the *i*^*th*^ round, the supplier alliance’s profit is *βα*^*i*−1^Δ*π* and the manufacturer’s profit is *α*^*i*^Δ*π*; and the profits of the suppliers are $\beta \alpha^{i-1}\Delta \pi /\eta_{\hbar }{+\pi }_{j}^{D*}$. By negotiating earlier, the supplier can obtain more profits $\beta (\alpha^{i-1}-\alpha^{\xi -1})\Delta \pi /\eta_{\hbar }$. However, supplier alliances must transfer profits to the manufacturer to negotiate in the *i*^*th*^ round. Denote the supplier alliance’s strategy as *ST*_*i*_ = (*F*_1_, *F*_2_,⋯,*F*_*i*_) (*i* = 1,2,⋯,*ξ*), where *F*_*i*_ is the profits that the supplier alliance needs to transfer. The supplier alliance’s profit in the *i*^*th*^ round is *βα*^*i*−1^Δ*π*. Therefore, the profits they can obtain are $\beta \alpha^{i-1}\Delta \pi /\eta_{\hbar }-F_{i}/\eta_{\hbar }{+\pi }_{j}^{D*}$. When supplier alliance negotiates in the last round, the profits of the supplier alliance are *βα*^*ξ*−1^Δ*π*, and the profits of suppliers are $\beta \alpha^{\xi -1}\Delta \pi /\eta_{\hbar }{+\pi }_{j}^{D*}$, which are the lowest profits obtained by supplier negotiation. Thus, *P*_*ξ*_ = 0. The transferred profits of supplier alliance are $\beta (\alpha^{i-1}-\alpha^{\xi -1})\Delta \pi$.

When the suppliers form multiple alliances to negotiate with the manufacturer, the alliance must give profits to the manufacturer to negotiate earlier. After giving profits, the final profits the suppliers can obtain are $\beta \alpha^{i-1}\Delta \pi /\eta_{\hbar }-F_{i}^{**}/\eta_{\hbar }{+\pi }_{j}^{D*}$. By substituting $F_{i}^{**}$ into the suppliers’ actual profits, it can conclude that after supplier alliances transfer profits, the suppliers can only obtain the profits of the last round of negotiations. The final profits are $\beta \alpha^{\theta -1}/\eta_{\hbar }+\pi_{j}^{D*}$, and the final profits of the manufacturer are $\pi_{m}^{**}=(1-\xi \beta \alpha^{\xi -1})\Delta \pi +\pi_{m}^{D*}$.

Q.E.D.

When the suppliers form multiple alliances to negotiate with the manufacturer, the supplier alliance can gain more profits by negotiating earlier. The suppliers’ profits are also affected by negotiation power, the number of supplier alliances, and the number of suppliers within the alliance. However, since the manufacturer has the right to decide on the negotiation order, the supplier alliance must give profits to the manufacturer to negotiate earlier; suppliers may obtain last-round profits and experience lower profits. Similarly, negotiation power and the number of supplier alliances affect the manufacturer’s profits, and the profits are the sum of its negotiation gains and the transferred profits of all supplier alliances. The manufacturer obtains the excess profits.

### 5.3 Suppliers form a grand alliance to negotiate with manufactures

We find that when suppliers form multiple alliances, they will still experience lower profits due to the competition between the alliances in the negotiation order. Therefore, suppliers consider forming a grand alliance to negotiate to reduce further the profit losses caused by the contention of the negotiation order.

When all suppliers form a grand alliance, considering that the grand alliance’s negotiating power is the average value of each alliance, the supplier alliance’s negotiating power is the average of the negotiating power of its members [52], i.e., $\beta_{B^{*}}=\frac{\sum_{\hbar =1}^{\xi }\beta_{B_{\hbar }}}{\xi }=\beta$. Furthermore, the manufacturer’s negotiating power with the grand alliance is $\alpha_{B^{*}}=\frac{\sum_{\hbar =1}^{\xi }\alpha_{B_{\hbar }}}{\xi }=\alpha$ and meets $\alpha_{B^{*}}+\beta_{B^{*}}=1$.

**Proposition 5.** All suppliers form a grand alliance, and the suppliers’ profits are $\pi_{j}^{***}=\beta \Delta \pi /n+\pi_{j}^{D*}$, and the manufacturer’s profits are $\pi_{m}^{***}=\alpha \Delta \pi +\pi_{m}^{D*}$.

*Proof*. If all suppliers form a grand alliance to negotiate, the profit distributions of the manufacturer and supplier alliance are *α*Δ*π* and *β*Δ*π*, respectively. Suppliers divide the supplier alliance’s profits equally, with each supplier’s profits being $\beta \Delta \pi /n+\pi_{j}^{D*}$ and the manufacturer’s profits being $\alpha \Delta \pi +\pi_{m}^{D*}$.

Q.E.D.

When all suppliers form a grand alliance, it can effectively resolve the excess profits gained by the manufacturer. The suppliers’ profits are affected by the supplier alliance’s negotiating power and the number of suppliers.

### 5.4 Supplier alliances strategy analysis

We consider that all suppliers are farsighted, and they can communicate with each other and freely form an alliance. Each supplier is free to join or leave the alliance or form a new alliance with other suppliers, and each supplier joins or leaves it to obtain more profits. When making alliance decisions, suppliers should consider the impact of their own alliance decisions on profits and the impact of other suppliers’ alliance decisions on profits, and the final alliance strategy should make all suppliers’ profits not lower than those under other alliance negotiation strategies.

In addition, when suppliers form more than one alliance, their profits are affected by the number of alliances and alliance members. We find that when the number of members of each alliance is not equal, the farsighted suppliers will join the alliance with fewer members to obtain a higher profit distribution, which eventually leads to the suppliers either choosing an independent alliance, choosing to form a grand alliance, or forming equal-sized alliances.

Next, we examine how supplier alliance strategies change to obtain more profits, considering the manufacturer’s negotiating power.

**Proposition 6**. When *α*<0.5, the negotiation power of the manufacturer is weaker than that of the supplier; all suppliers form a grand alliance and can gain more profits.

*Proof*. When all suppliers form a grand alliance, from Proposition 5 that the suppliers’ profits are $\pi_{j}^{***}=\beta \Delta \pi /n+\pi_{j}^{D*}$. When suppliers form independent alliances, from Proposition 3 that the suppliers’ profits are $\pi_{j}^{*}=\beta \alpha^{n-1}\Delta \pi +\pi_{j}^{D*}$. When suppliers form *ξ* equal-sized alliances, the suppliers’ profits are πj**=βαξ−1nξΔπ+πjD*.

Comparing $\pi_{j}^{***}$ and $\pi_{j}^{**},\;\pi_{j}^{***}$ and $\pi_{j}^{*}$, respectively. we can get that when $\alpha <({\frac{1}{\xi })}^{\frac{1}{\xi -1}}(\xi >1)$, $\pi_{j}^{***}>\pi_{j}^{**}$; and when $\alpha <({\frac{1}{n})}^{\frac{1}{n-1}}(n>1)$, $\pi_{j}^{***}>\pi_{j}^{*}$.

**Fig 1:**
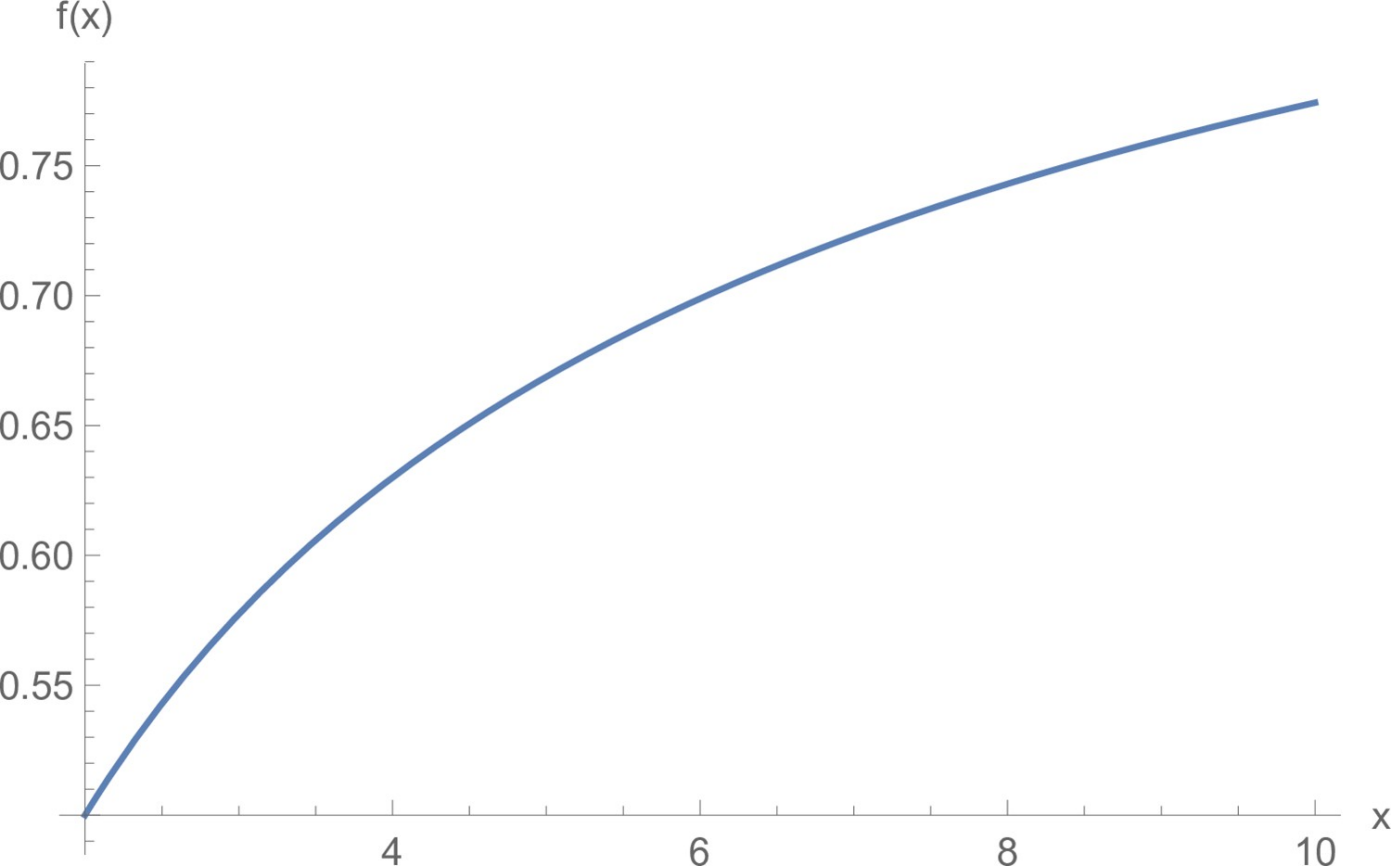
The function image of *f*(*x*).

Set $f(x)=({\frac{1}{x})}^{\frac{1}{x-1}}(x>1)$. As shown in the graph (see Fig 1), *f*(*x*) monotonically increases for (1, +∞). Since when *ξ*≤*n*, $({\frac{1}{\xi })}^{\frac{1}{\xi -1}}<({\frac{1}{n})}^{\frac{1}{n-1}}$ is always true. Thus, when the manufacturer’s negotiation power meets $\alpha <({\frac{1}{\xi })}^{\frac{1}{\xi -1}}(\xi >1)$, $\alpha <({\frac{1}{n})}^{\frac{1}{n-1}}(n>1)$ is always true. So, when $\alpha <({\frac{1}{\xi })}^{\frac{1}{\xi -1}}(\xi >1)$, $\pi_{j}^{***}>\pi_{j}^{**}$ and $\pi_{j}^{***}>\pi_{j}^{**}$ are founded at the same time. For suppliers, forming a grand alliance could result in more profits.

In addition, because of $f(x)=({\frac{1}{x})}^{\frac{1}{x-1}}(x>1)$ monotone increasing, so the $({\frac{1}{\xi })}^{\frac{1}{\xi -1}}$ increases with the increase of *ξ*. When *ξ*>1, deduced the minimum can take to 2, at this time, $({\frac{1}{\xi })}^{\frac{1}{\xi -1}}$ also takes the minimum value, i.e. the $({\frac{1}{2})}^{\frac{1}{2-1}}$ is equal to 0.5. Combined with the previous assumption *α*+*β* = 1, therefore, when *α*<0.5, the manufacturer’s negotiating power is weaker than that of the supplier. Therefore, it can prove that Proposition 6 is true; that is, when α<0.5, the negotiation power of the manufacturer is weaker than that of the supplier; all suppliers form a grand alliance and can gain more profits.

Q.E.D.

If the manufacturer’s negotiating power is weak, the best choice for suppliers is to form a grand alliance to negotiate, which can increase profits.

**Proposition 7.** When $\frac{\varepsilon -1}{\varepsilon }<\alpha <\frac{\varepsilon }{\varepsilon +1}$, suppliers can obtain more profits by forming *ε* equal-sized alliances. Moreover, the stronger the manufacturer’s negotiating power is, the more equal-sized alliances there are, and the more profits suppliers can obtain.

**Fig 2:**
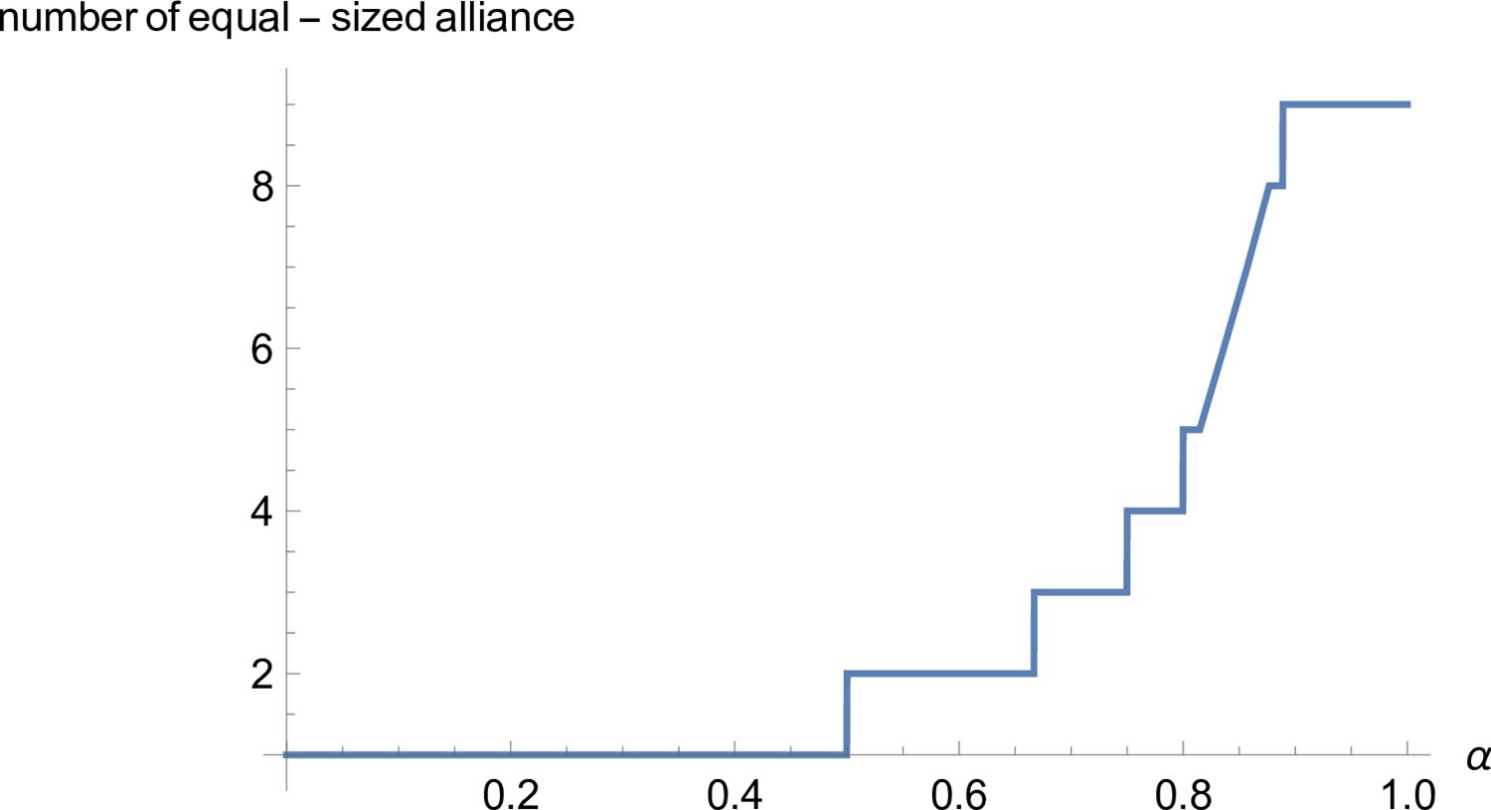
Relationship between manufacturers’ negotiating power and the number of equal-sized alliances.

*Proof*. When there are *ε*(1<*ε*<*n*) equal-sized alliances, the number of members of each alliance is nε and meets *n mod ε* = = 0, and the profit distribution of each supplier is βαε−1nε. Suppliers form a grand alliance, and the suppliers’ profits are $\frac{\beta }{n}$. When *ε* = 2, the supplier forms two equal-sized alliances, and the suppliers’ profits are βαn2. When *ε* = 3, the supplier forms three equal-sized alliances, and the suppliers’ profits are βαn3. When the manufacturer’s negotiating power meets 12<α<23,βn<βαn2 and βαn2>βα2n3 are established simultaneously, and the suppliers can obtain the most profits by forming two equal-sized alliances. When the manufacturer’s negotiating power meets 23<α<34,βαn2<βα2n3 and βα2n3>βα3n4 are established simultaneously, and the suppliers can obtain the most profits by forming three equal-sized alliances. It can prove that if the manufacturer’s negotiating power meets $\frac{\varepsilon -1}{\varepsilon }<\alpha <\frac{\varepsilon }{\varepsilon +1}$, the supplier can obtain more profits by forming *ε* equal-sized alliances. We take the eight suppliers as an example and further illustrate it (see Fig 2).

As shown in Fig 2, when the manufacturer’s negotiating power meets $\frac{\varepsilon -1}{\varepsilon }<\alpha <\frac{\varepsilon }{\varepsilon +1}$, as the manufacturer’s negotiating power strengthens, the more equal-sized alliances there are, and the more profits suppliers can obtain.

Q.E.D.

When $\alpha >({\frac{1}{n})}^{\frac{1}{n-1}}$, $\pi_{i}^{*}>\pi_{i}^{***}$. When $\alpha >({\frac{\varepsilon }{n})}^{\frac{1}{n-\varepsilon }},\;\pi_{j}^{*}>\pi_{j}^{**}$. Therefore, when $\alpha >Max\{({\frac{1}{n})}^{\frac{1}{n-1}},{\left(\frac{\varepsilon }{n}\right)}^{\frac{1}{n-\varepsilon }}\},\;\pi_{j}^{*}>\pi_{j}^{***}$ and $\pi_{j}^{*}>\pi_{j}^{**}$ are established at the same time, the profits obtained by suppliers forming independent alliances are higher than those obtained by forming *ξ* equal-sized alliances or grand alliances, and the best choice for suppliers is to form independent alliances.

**Proposition 8.** When $\alpha >Max\{({\frac{1}{n})}^{\frac{1}{n-1}},\;{\left(\frac{\varepsilon }{n}\right)}^{\frac{1}{n-\varepsilon }}\}$, suppliers can obtain more profits by forming independent alliances.

***Proof*.** When all suppliers form a grand alliance, the suppliers `profits are $\pi_{j}^{***}=\beta \Delta \pi /n+\pi_{j}^{D*}$. When suppliers form independent alliances, the suppliers`profits are $\pi_{j}^{*}=\beta \alpha^{n-1}\Delta \pi +\pi_{j}^{D*}$. When suppliers form ξ equal-sized alliances, the profits of suppliers are πj**=βαξ−1nξΔπ+πjD*.

Q.E.D.

When $\alpha >({\frac{1}{n})}^{\frac{1}{n-1}}$, $\pi_{i}^{*}>\pi_{i}^{***}$, and when $\alpha >({\frac{\varepsilon }{n})}^{\frac{1}{n-\varepsilon }}$, $\pi_{j}^{*}>\pi_{j}^{**}$. Therefore, when $\alpha >Max\{({\frac{1}{n})}^{\frac{1}{n-1}},\;{\left(\frac{\varepsilon }{n}\right)}^{\frac{1}{n-\varepsilon }}\},\;\pi_{j}^{*}>\pi_{j}^{***}$ and $\pi_{j}^{*}>\pi_{j}^{**}$ are established at the same time, the profits obtained by suppliers forming independent alliances are higher than those obtained by forming *ξ* equal-sized alliances or grand alliances, and the best choice for suppliers is to form independent alliances.

### 6. Numerical analysis

We conducted a correlation analysis using the following calculation examples to visualize the influence of parameter values on optimal strategy.

In 2020, The General Office of the State Council issued the “New Energy Vehicle Industry Development Plan (2021–2035)” [53] and set the goal that NEVs sales will reach approximately 20% of the total automobile sales by 2025. Based on 25.31 million vehicle sales in 2020, the market size of NEVs will exceed 5 million units, set *a* = 5×10^6^.

Based on the calculation method of NEVs credit in the dual credit policy, which was issued in 2020 [5], the credit of each pure electric passenger vehicle = (0.0056×range +0.4) × power consumption adjustment coefficients × battery energy density adjustment coefficients, set the values *λ* = 0.006.

In May 2021, the Ministry of Industry and Information Technology issued “The Annual Report on the Implementation of Parallel Management of Average Fuel Consumption of Passenger Vehicle Enterprises and New Energy Vehicles Credits (2021)”, which showed that the average credit price is 1204, and the value, set *p*_*e*_ = 1200.

According to the reference [54], set *θ* = 100.

The following analysis is carried out through an numerical example to visually demonstrate the impact of different parameter values on optimal strategies. For example, we use six parts suppliers to perform numerical analysis.

Meeting the assumptions, we use an arbitrarily chosen numerical experiment for the other parameters to verify our model. The other relevant parameters are *k*_1_ = 300000, *k*_2_ = 500000, *k*_3_ = 1000000, *k*_4_ = 400000, *k*_5_ = 500000, *k*_6_ = 600000, *k*_*m*_ = 500000, *α* = 0.4, and *β* = 0.6.

The alliance structure formed by six suppliers is an independent alliance $\bar{B}=\{\{1\},\{2\},\{3\},\{4\},\{5\},\{6\}\}$, grand alliance ℬ* = {1,2,3,4,5,6}. Suppliers forming equal-sized alliances is complicated, and six suppliers can freely form different equal-sized alliances. For example, supplier can form three equal-sized alliances, such as the {{1, 2}, {3, 4}, {5, 6}}, {{1, 3}, {2, 4}, {5, 6}}, {{1, 4}, {2, 3}, {5, 6}}, {{1, 5}, {3, 4}, {2, 6}}, {{1, 6}, {3, 4}, {2, 6}}, etc. Suppliers can also form two equal-sized alliances, such as the {{1,2,3}, {4,5,6}}, {{1,2,4}, {3,5,6}}, {{1,2,5}, {3,5,6}}, {{1,2,6}, {3,5,6}}, etc. Combined with the previous Proposition, when suppliers form equal-sized alliances, the negotiating power and the number of equal-sized alliances affect the suppliers’ profits simultaneously. Since suppliers have the same negotiating power, the suppliers’ profits are only related to the number of equal-sized alliances. For simplicity, we use an arbitrarily chosen equal-sized alliance, namely {{1,2}, {3,4}, {5,6}}, {{1,2,3}, {4,5,6}}, to verify three equal-sized alliances and two equal-sized alliances’ impact on suppliers’ profits.

We Substitute these parameters into the optimal technological innovation strategy for NEVs, shown in Table 1.

**Table 1:** The manufacturer and suppliers’ technological innovation.

	Decentralized decisions	Centralized decisions
manufacturer	78	585
supplier 1	122	975
supplier 2	73	585
supplier 3	36	292
supplier 4	91	731
supplier 5	73	585
supplier 6	61	487

From Table 1, we can see that decentralized decision-making leads to dysfunctional decision-making in the NEVs supply chain due to the dual marginal effect, which decreases the technological innovation of manufacturers and suppliers.

Secondly, we discuss the NEVs supply chain cooperation through negotiations.

From Table 2, the results show that after suppliers distribute incremental profits through bargaining negotiation, the profits of NEV supply chain members are all higher than those in decentralized decision-making. The coordination strategy based on the negotiation game achieves a win-win situation for all parties, but the suppliers can obtain extra profits under different alliance strategies.

**Table 2:** The manufacturer and suppliers’ profits.

	Decentralized decisions	Centralized decisions			
		Alliance structure			
		{{1},{2},{3},{4},{5},{6}}	{{1,2}, {3,4}, {5,6}}	{{1,2,3}, {4,5,6}}	{1,2,3,4,5,6}
supplier 1	1.308×10^11^	1.622×10^11^	3.764×10^11^	5.401×10^11^	6.428×10^11^
supplier 2	1.317×10^11^	1.631×10^11^	3.773×10^11^	5.410×10^11^	6.433×10^11^
supplier 3	1.324×10^11^	1.638×10^11^	3.779×10^11^	5.417×10^11^	6.440×10^11^
supplier 4	1.314×10^11^	1.628×10^11^	3.769×10^11^	5.407×10^11^	6.430×10^11^
supplier 5	1.317×10^11^	1.631×10^11^	3.773×10^11^	5.410×10^11^	6.433×10^11^
supplier 6	1.319×10^11^	1.634×10^11^	3.775×10^11^	5.412×10^11^	6.436×10^11^

### 6.1 The influence of the dual credit policy

1) The influence of the credit factor on technological innovation

**Fig 3:**
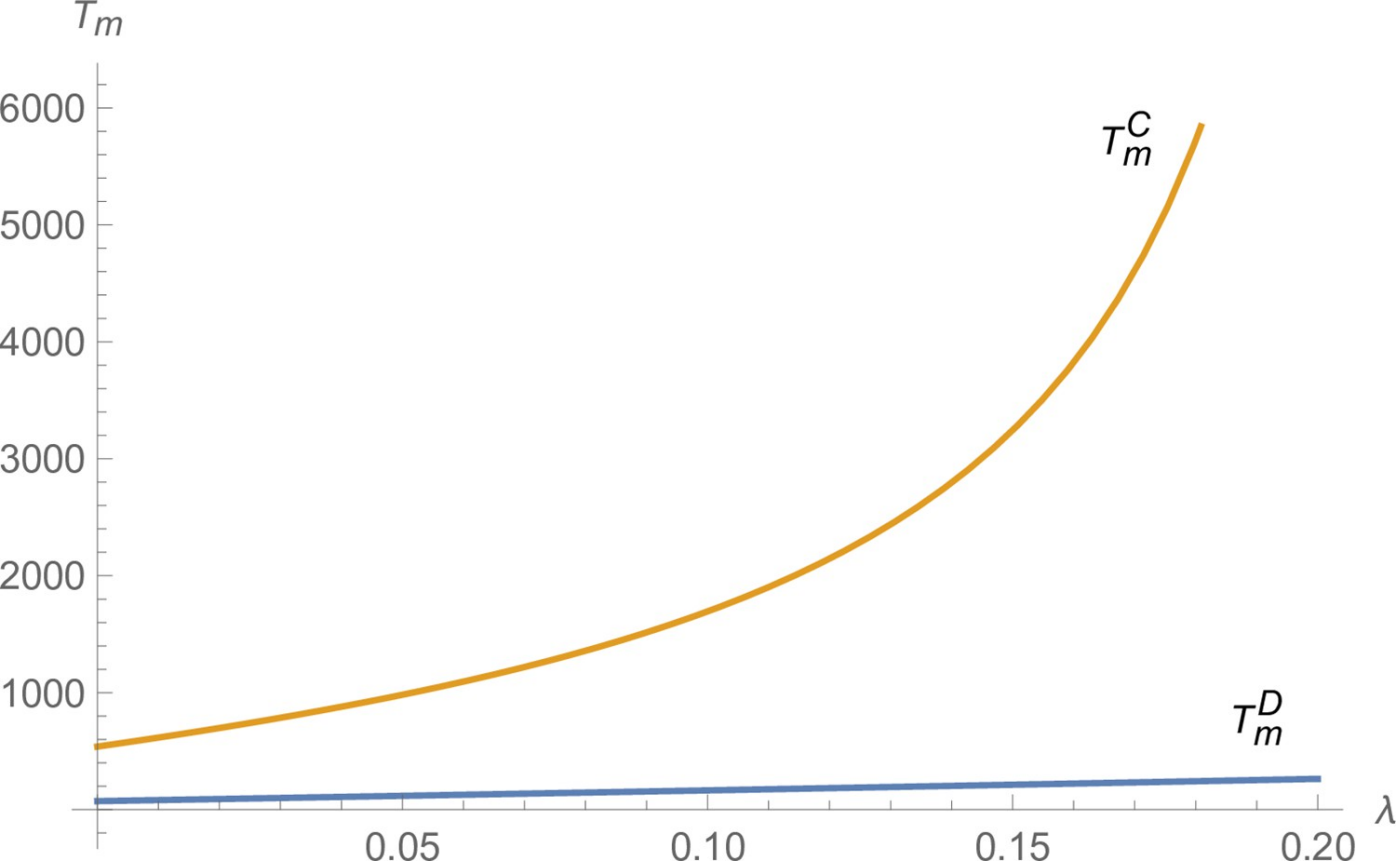
The impact of *λ* on *T*_*m*_.

**Fig 4:**
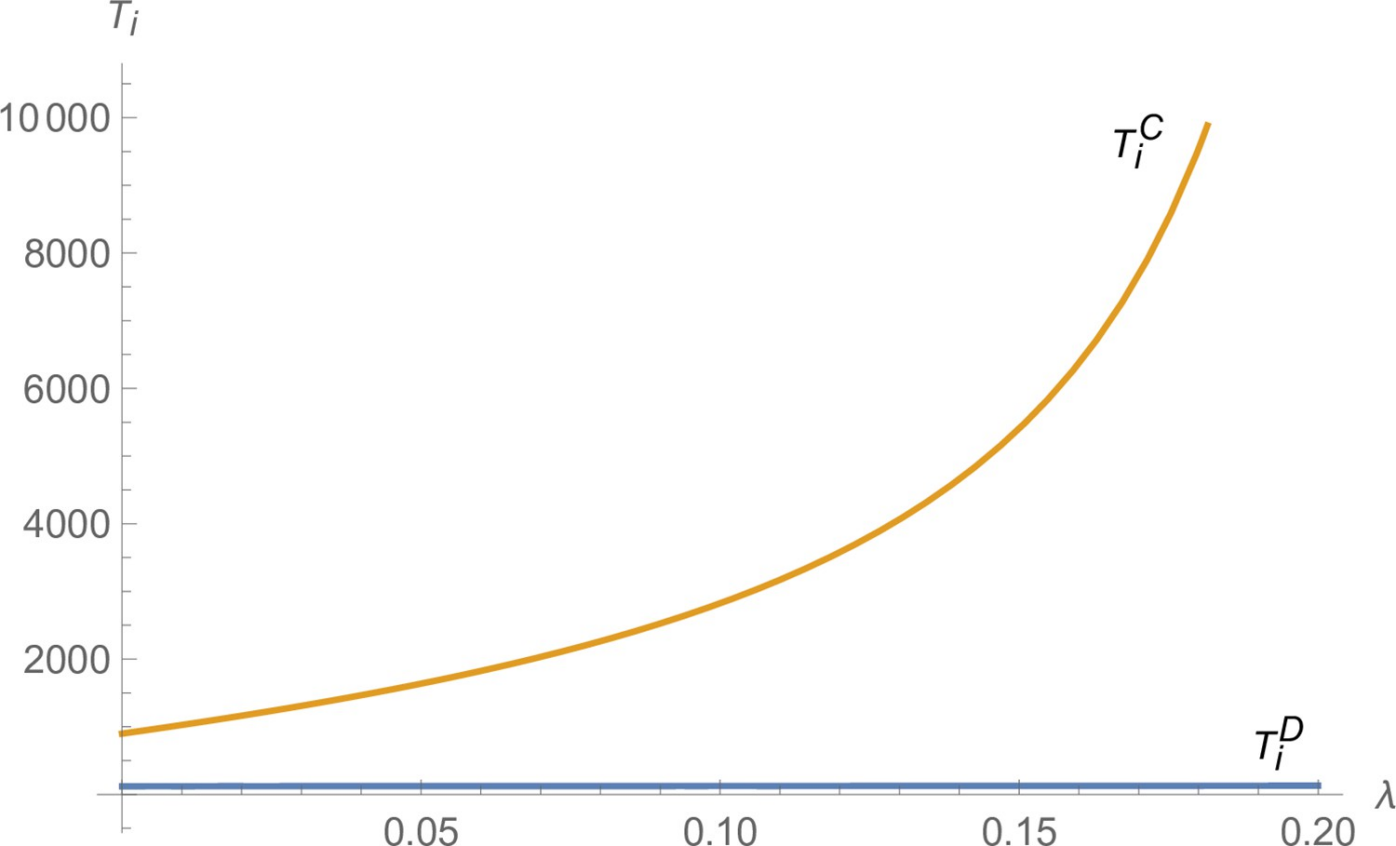
The impact of *λ* on *T*_*i*_.

**Fig 5:**
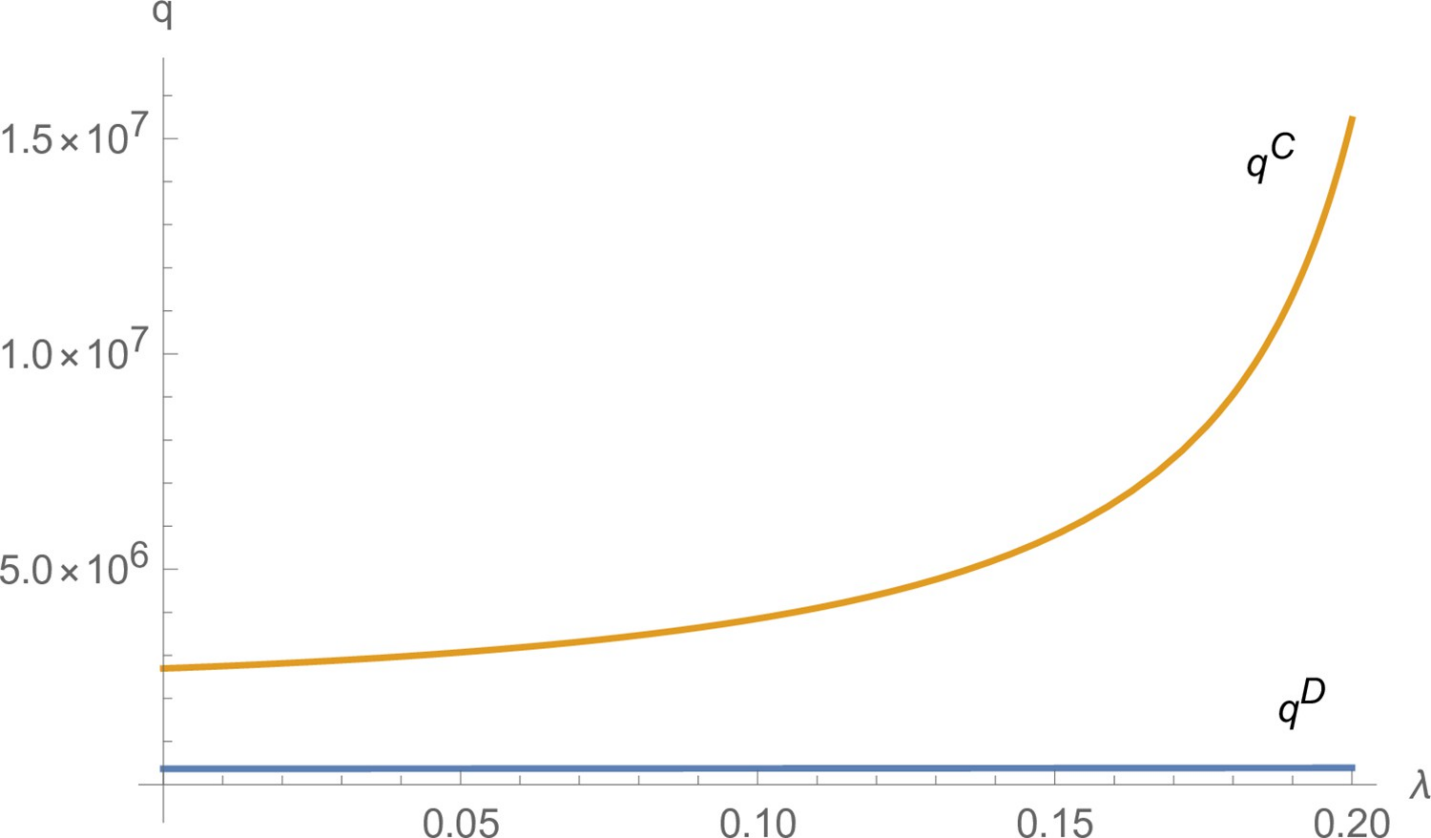
The impact of *λ* on *q*.

**Fig 6:**
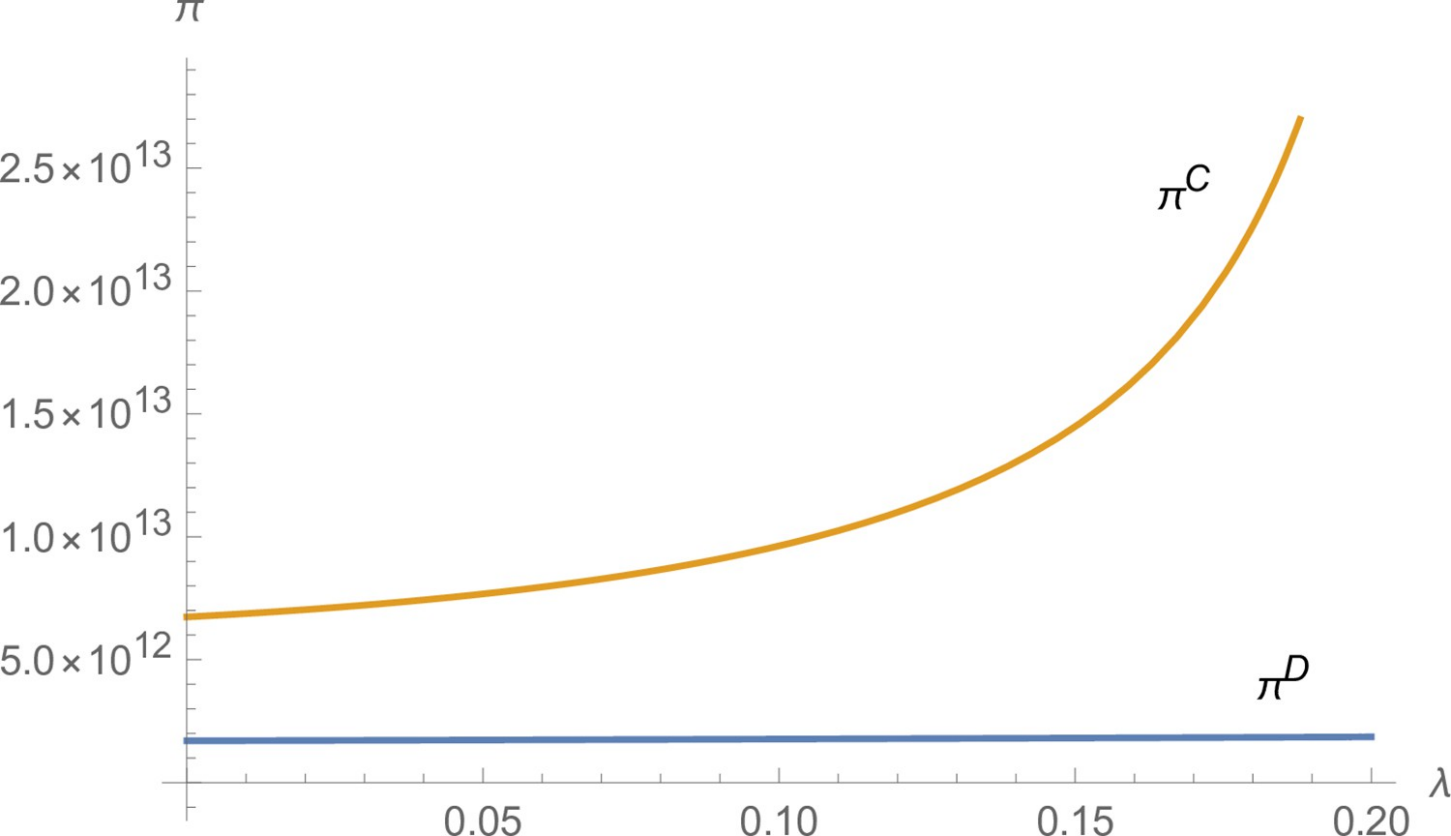
The impact of *λ* on *π*.

From Figs 3 and 4, the technological innovation of manufacturers and suppliers increases under centralized decision-making compared to decentralized decision-making, and the advantage of centralized decision-making increases as *λ* increases. From Fig 5, NEVs’ production under centralized decisions is far more than that under decentralized decisions and increases with *λ* increases. From Fig 6, the profits of the NEVs supply chain under centralized decisions are much higher than that under decentralized decisions and increase with *λ* increases.

Therefore, the policy has improved the technological innovation of NEVs, realized the NEVs’ large-scale development, and increased the profits of the NEVs supply chain. Decentralized decision-making has led to dysfunctional decision-making in the NEVs supply chain, and there is an urgent need to coordinate the NEVs supply chain to achieve optimal strategies under centralized decision-making.

The government can guide the credit factor on technological innovation and guide the NEVs supply chain to choose the centralized decision-making, thus promoting the development of the NEVs industry.

2) The influence of the credit price

**Fig 7:**
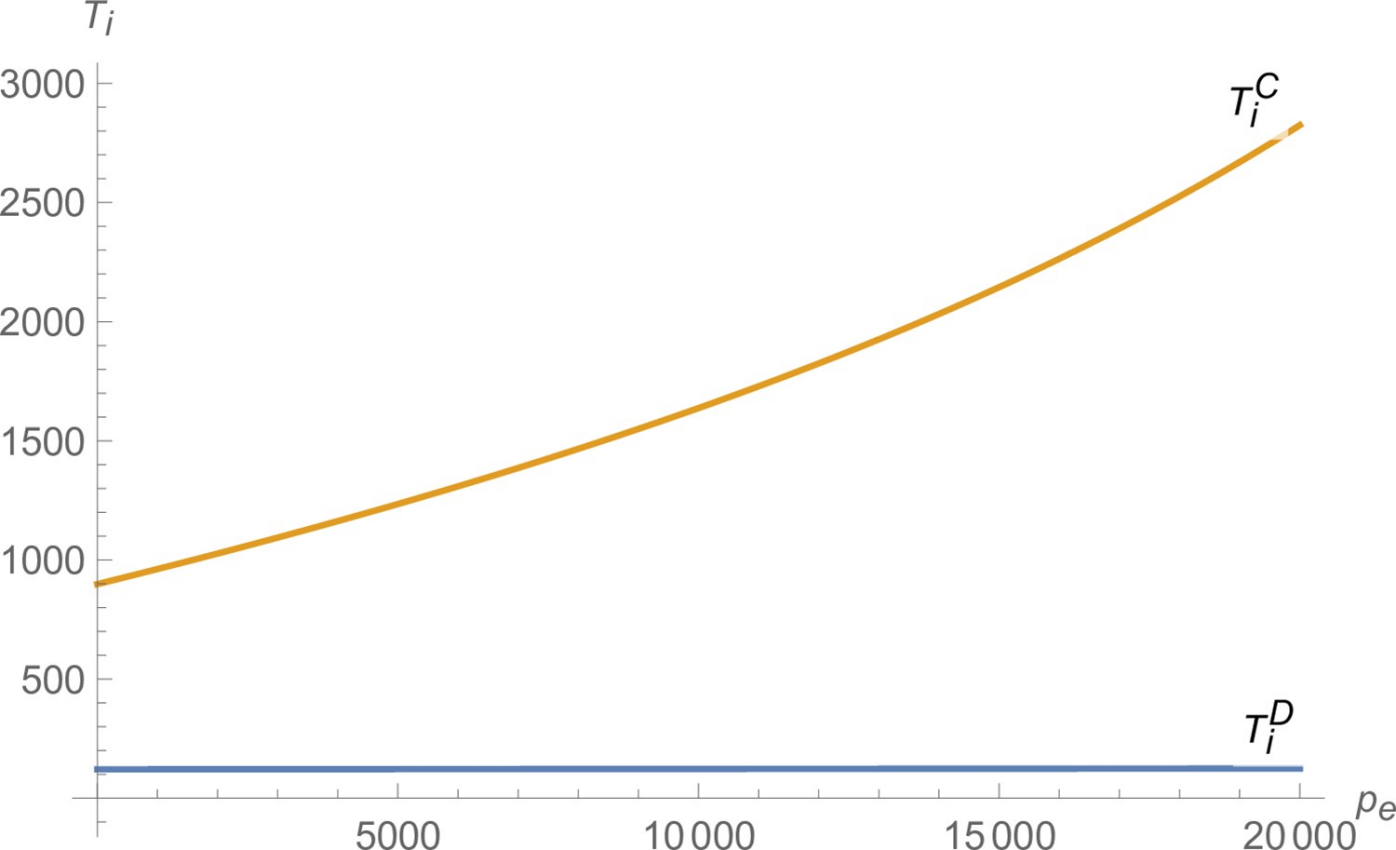
The impact of *p*_*e*_ on *T*_*i*_.

**Fig 8:**
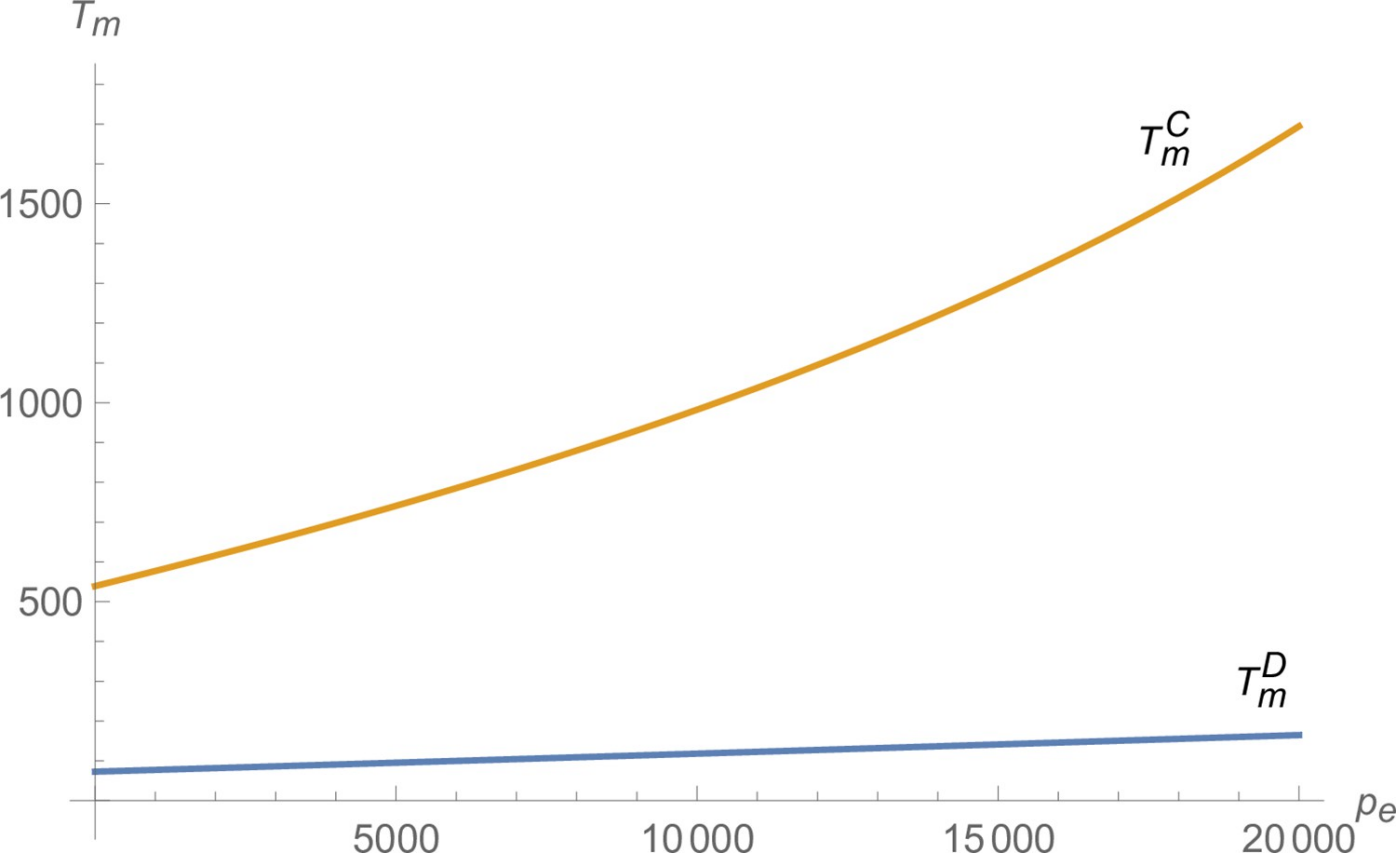
The impact of *p*_*e*_ on *T*_*m*_.

**Fig 9:**
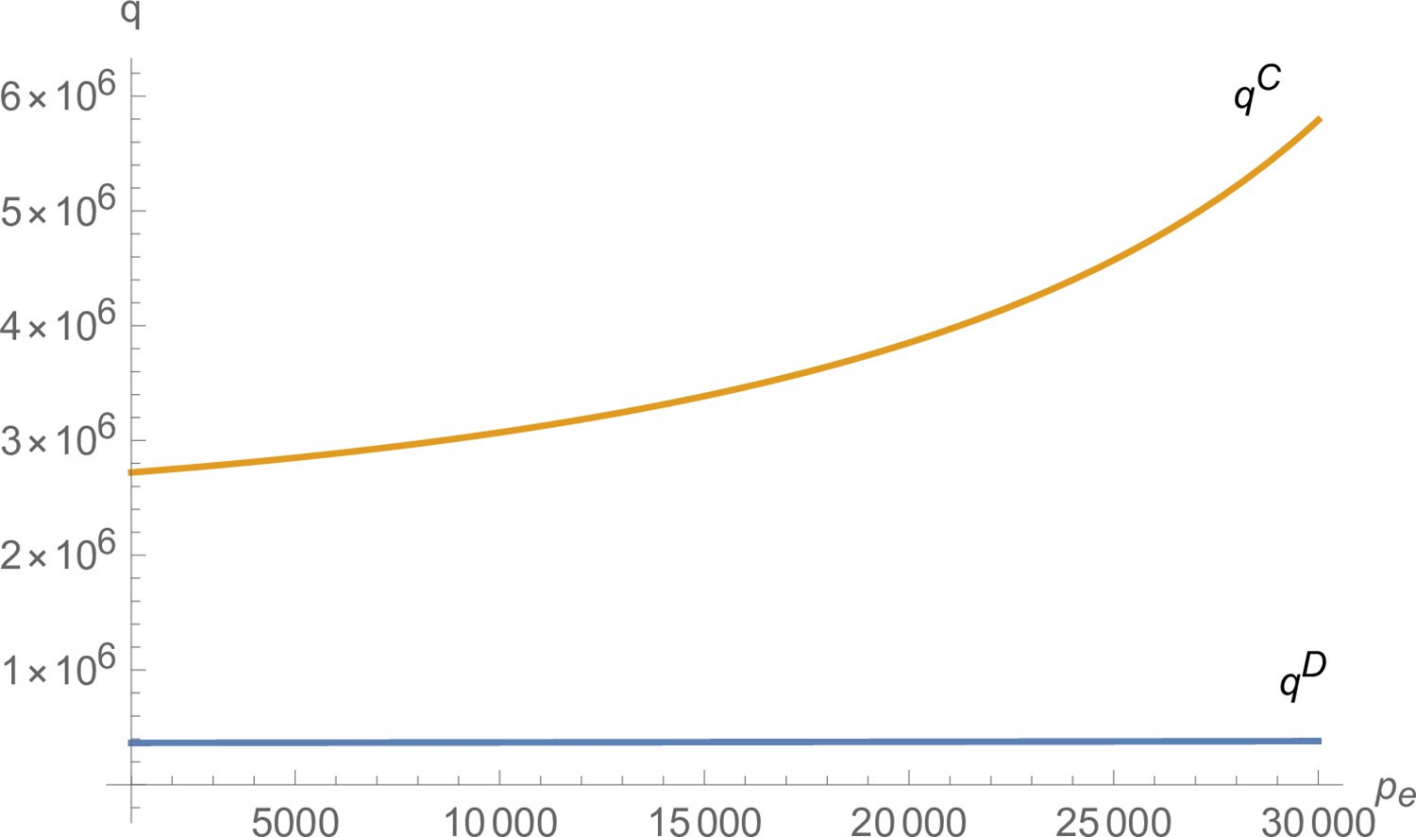
The impact of *p*_*e*_ on *q*.

**Fig 10:**
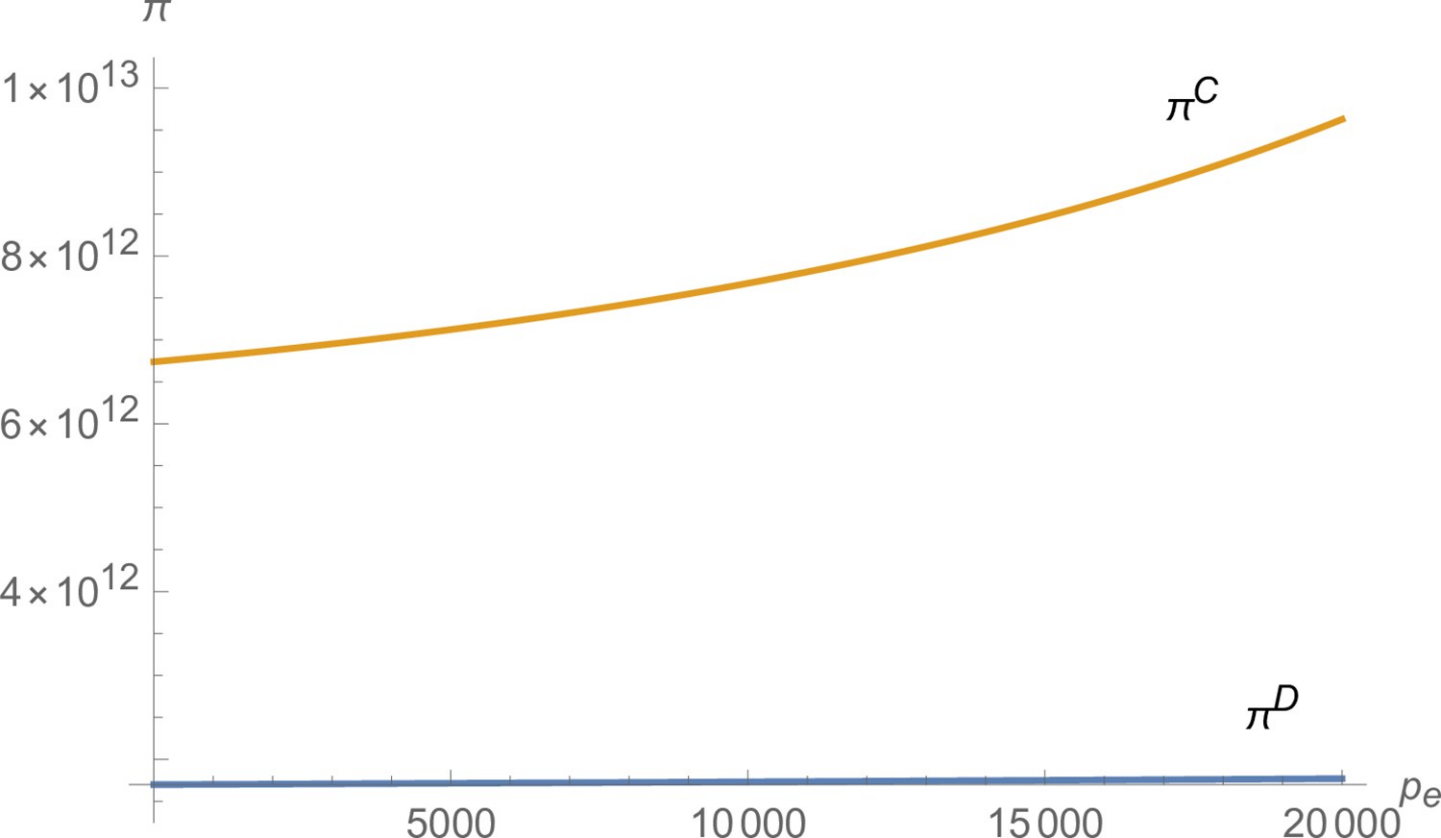
The impact of *p*_*e*_ on *π*.

From Figs 7 and 8, both manufacturer and suppliers’ technological innovation increases under centralized decision-making, and the advantage of centralized decision-making increases as *p*_*e*_ increases. From Fig 9, the production of NEVs under centralized decision-making is much larger than that under decentralized decision-making and increases with *p*_*e*_. From Fig 10, the profit of the NEVs supply chain under centralized decision-making is much higher than that under decentralized decision-making and increases with *p*_*e*_.

Therefore, the policy has promoted the improvement of the NEVs’ technological innovation, realized the rapid growth of NEVs, and increased the profit of the NEVs supply chain. Decentralized decision-making leads to dysfunctional decision-making in the NEVs supply chain, and there is an urgent need to adjust the NEVs supply chain to achieve the optimal strategy under centralized decision-making.

The government can guide the credit price and the NEVs supply chain to make centralized decisions, thus promoting the development of the NEVs industry.

### 6.2 Influence of the alliance negotiation

**Fig 11:**
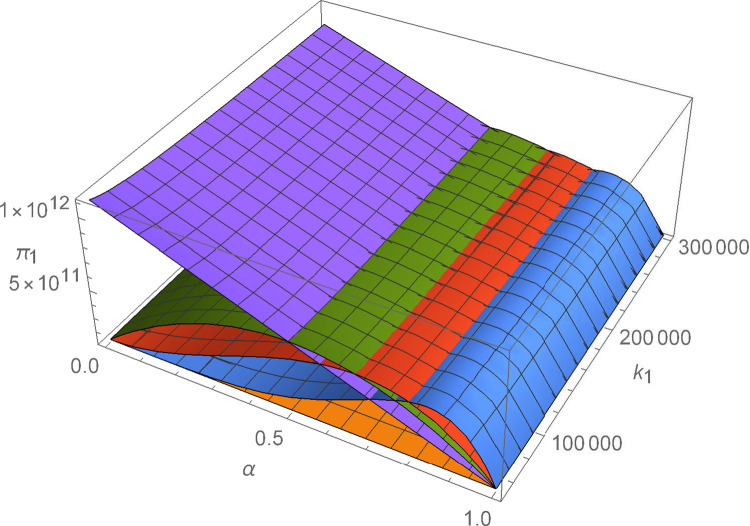
The impact of α and *k*_1_ on *π*_1_.

**Fig 12:**
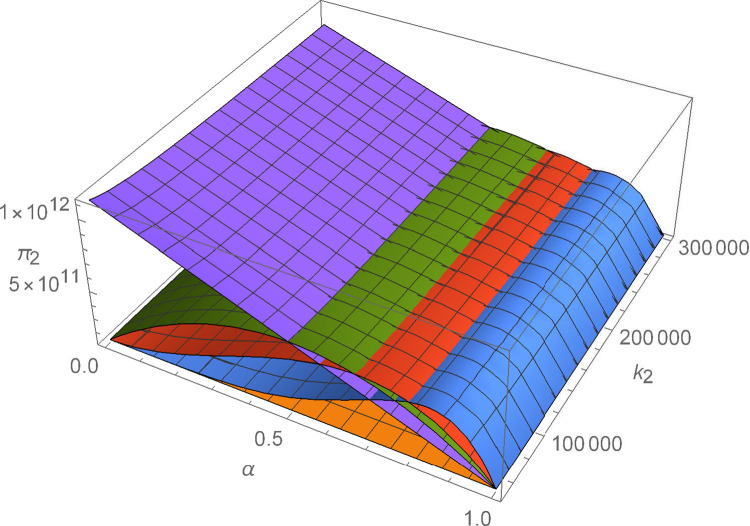
The impact of α and *k*_2_ on *π*_2_.

**Fig 13:**
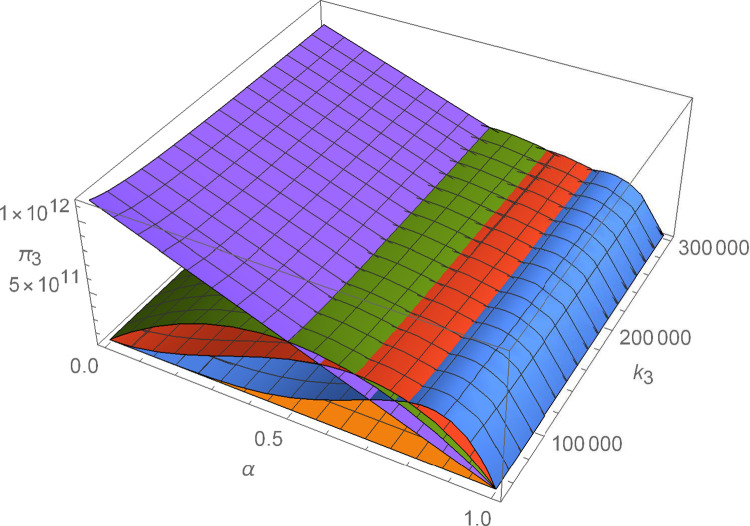
The impact of α and *k*_3_ on *π*_3_.

**Fig 14:**
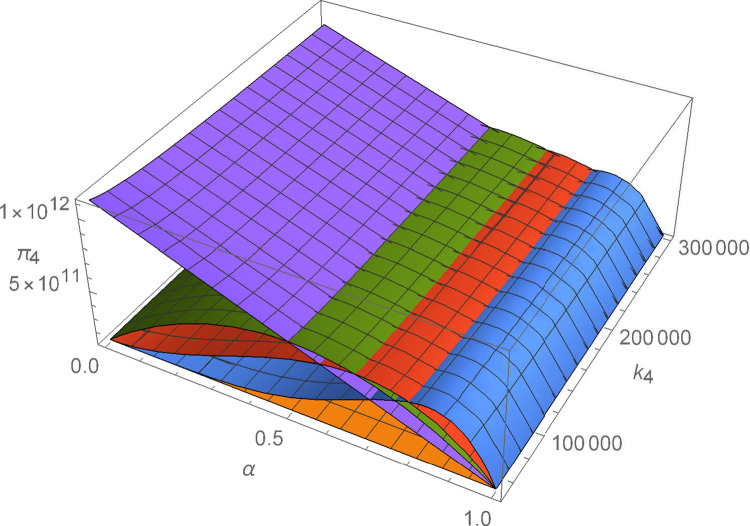
The impact of α and *k*_4_ on *π*_4_.

**Fig 15:**
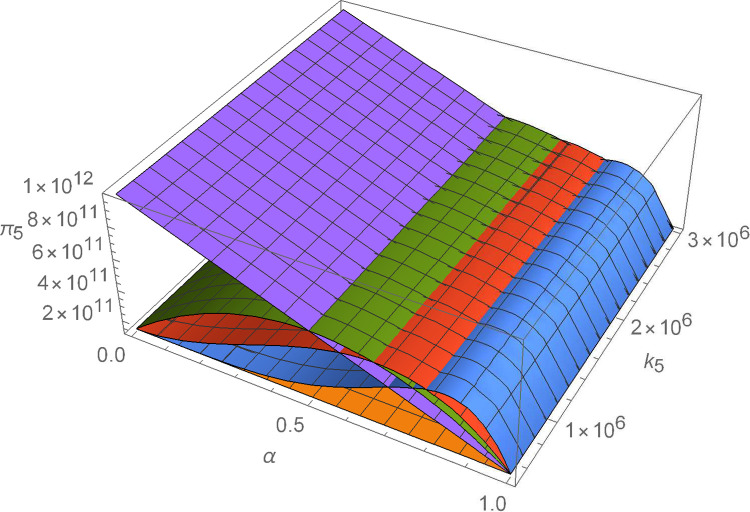
The impact of α and *k*_5_ on *π*_5_.

**Fig 16:**
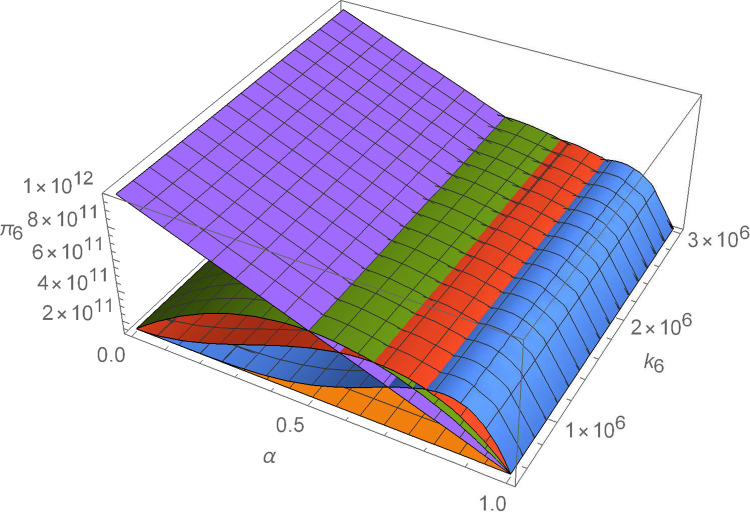
The impact of α and *k*_6_ on *π*_6_.

Figs 11–16 shows how suppliers’ profit varies with negotiating power and the cost coefficient of technological innovation. From Fig 11, no matter how the cost coefficient of technological innovation of supplier 1 changes, compared with decentralized decision-making, the profit-sharing mechanism based on centralized decision-making and alliance negotiation enables supplier 1 to obtain more profits. Meanwhile, with the change in negotiation power, supplier 1 can obtain different profits by choosing different alliance strategies. From Fig 12, no matter how the cost coefficient of technological innovation of supplier 2 changes, compared with decentralized decision-making, the profit-sharing mechanism based on centralized decision-making and alliance negotiation enables supplier 2 to obtain more profits. Meanwhile, with the change in negotiation power, supplier 2 can obtain different profits by choosing different alliance strategies. From Fig 13, no matter how the cost coefficient of technological innovation of supplier 3 changes, compared with decentralized decision-making, the profit-sharing mechanism based on centralized decision-making and alliance negotiation enables supplier 3 to obtain more profits. Meanwhile, with the change in negotiation power, supplier 3 can obtain different profits by choosing different alliance strategies. From Fig 14, no matter how the cost coefficient of technological innovation of supplier 4 changes, compared with decentralized decision-making, the profit-sharing mechanism based on centralized decision-making and alliance negotiation enables supplier 4 to obtain more profits. Meanwhile, with the change in negotiation power, supplier 4 can obtain different profits by choosing different alliance strategies. From Fig 15, no matter how the cost coefficient of technological innovation of supplier 5 changes, compared with decentralized decision-making, the profit-sharing mechanism based on centralized decision-making and alliance negotiation enables supplier 5 to obtain more profits. Meanwhile, with the change in negotiation power, supplier 5 can obtain different profits by choosing different alliance strategies. From Fig 16, no matter how the cost coefficient of technological innovation of supplier 6 changes, compared with decentralized decision-making, the profit-sharing mechanism based on centralized decision-making and alliance negotiation enables supplier 6 to obtain more profits. Meanwhile, with the change in negotiation power, supplier 6 can obtain different profits by choosing different alliance strategies.

### 6.3 The influence of negotiating power

**Fig 17:**
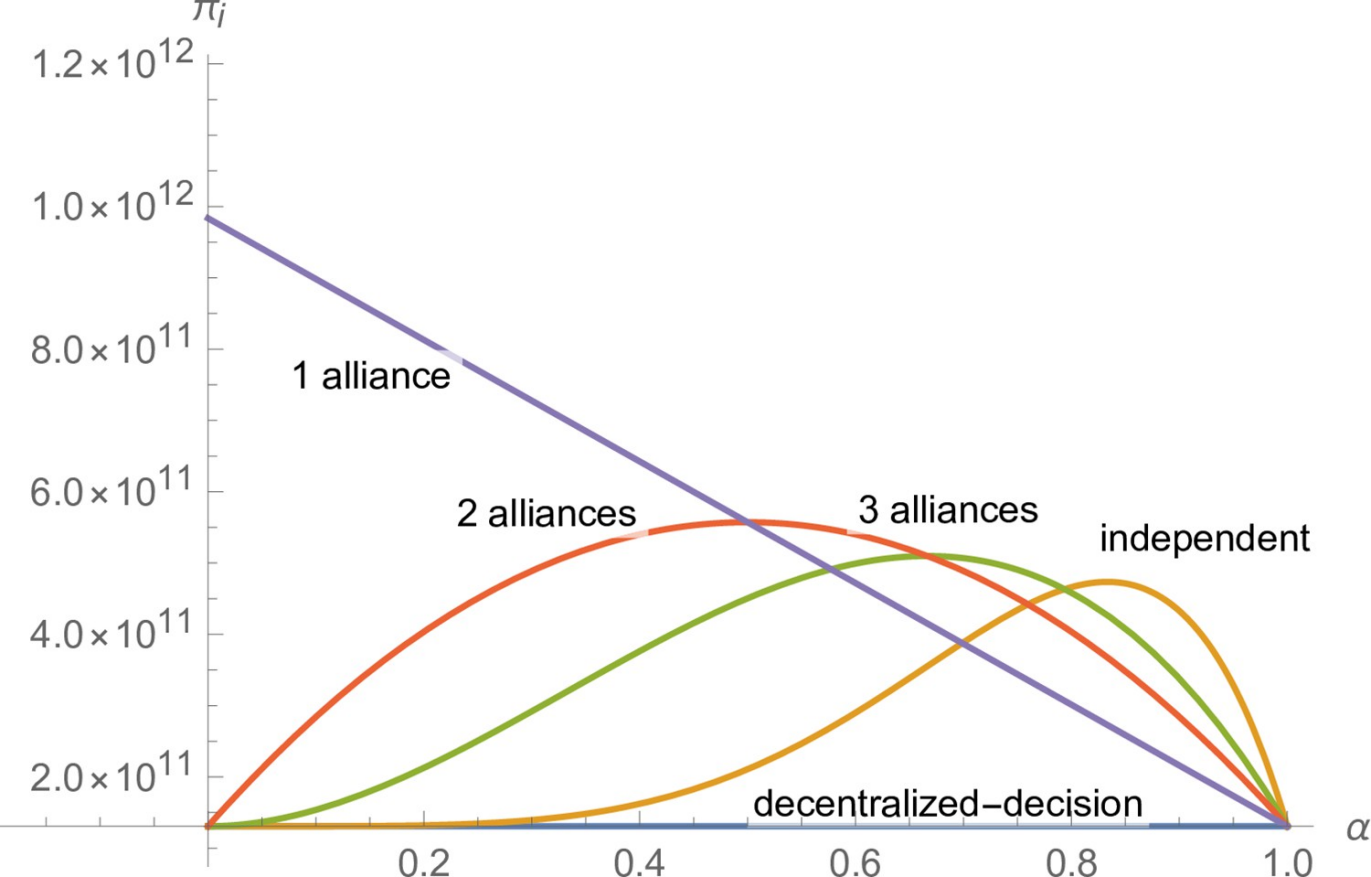
The impact of α on *π*_*j*_.

**Fig 18:**
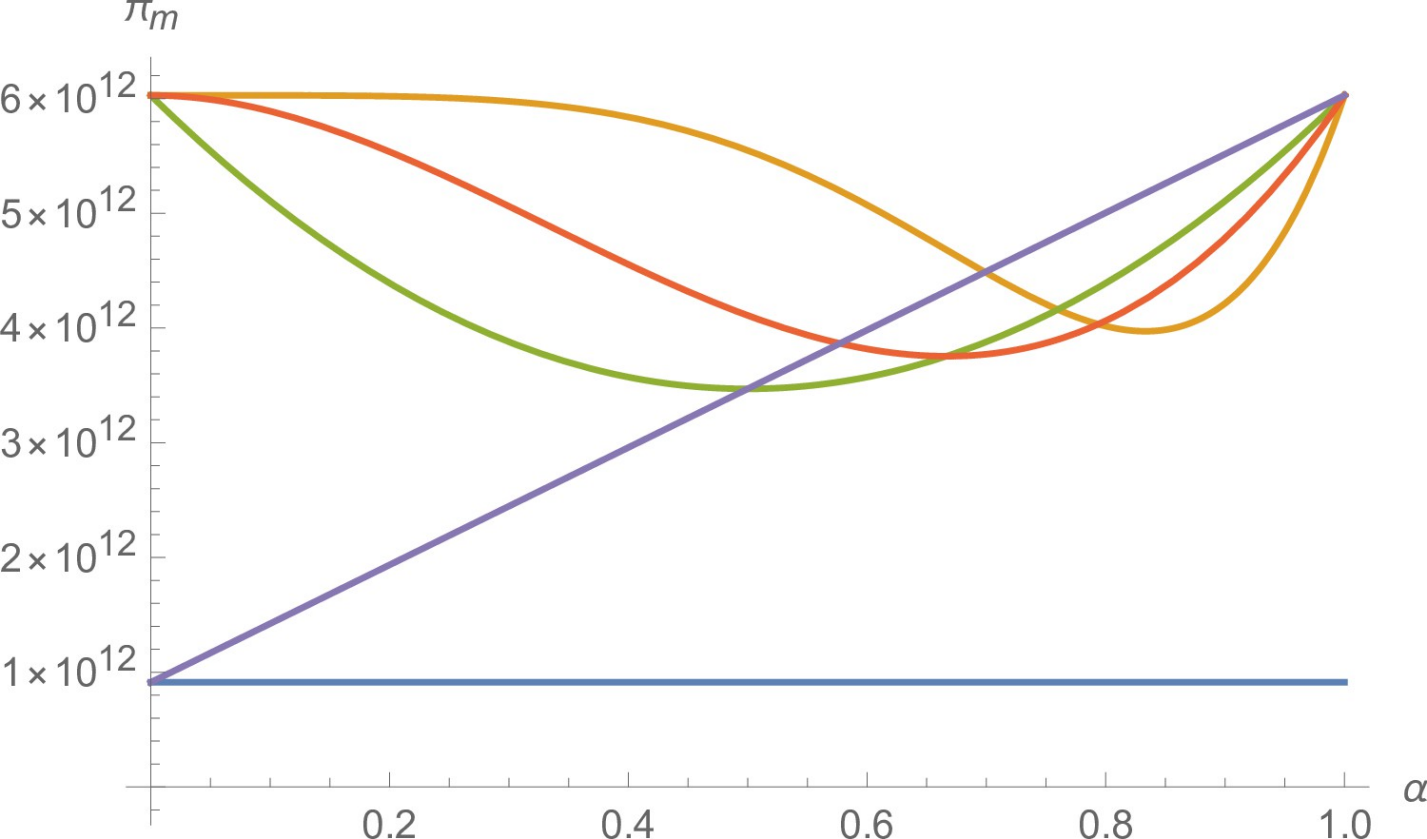
The impact of α on *π*_*m*_.

From Figs 17 and 18, establishing a coordination mechanism based on the negotiation game model improves the profits of both suppliers and manufacturers over decentralized decision-making.

From Fig 17, negotiating power and suppliers’ alliance strategy affect suppliers’ profits. With different negotiating power, suppliers’ alliance strategies will also impact profits. By forming a grand alliance, suppliers can obtain higher profit distribution when the manufacturer’s negotiating power is weak. With the manufacturer’s negotiating power increasing, the supplier’s profits decrease, but at this time, the suppliers can obtain the maximum profit distribution by forming two equal-sized alliances. Suppliers can obtain the maximum profit distribution as the negotiating power increases by forming three equal-sized alliances. When the manufacturer’s negotiating power reaches a certain threshold, suppliers can obtain the maximum profit distribution by forming an independent alliance.

From Fig 18, when the manufacturer’s negotiating power improves, the manufacturer’s profits will increase. Since the distributable profit increment is certain, the manufacturer’s profits will change in the opposite direction of the suppliers’ profits, but it is still much higher than those under decentralized decisions.

## 7 Conclusions and policy recommendations

### 7.1 Conclusions

In this paper, we construct decentralized and centralized decision models under the dual credit policy and obtain the optimal technology innovation strategy for the NEVs supply chain. Furthermore, we construct bargaining game models based on supplier alliances to distribute the supply chain’s incremental profits and study the supply chain’s coordination strategy. In addition, we discuss the suppliers’ proper alliance strategy for getting more profits under the manufacturer’s different bargaining power.

We found that the higher the credit factor of technology innovation is, the higher the credit price and the higher the technology innovation investment of the supply chain. The policy can effectively stimulate the Nevs’ supply chain to increase technology innovation investment, improve the NEVs’ technical level, and improve the profits of the supply chain.

The bargaining game model based on the supply chain alliance coordinates the NEVs supply chain so that the independent supply chain enterprises can realize the optimal strategy under the centralized decision. Suppliers can choose the best alliance strategy to improve their profits under the manufacturer’s different negotiating power.

When the manufacturer’s negotiating power is weak, suppliers can gain more profits by forming a grand alliance. When the manufacturer’s negotiating power is within a specific range, the suppliers form equal-sized alliances to participate in the negotiation. Moreover, as the negotiating power strengthens, the more suppliers form equal alliances, the more profits they can obtain. When the manufacturer’s negotiating power increases to a certain threshold, the supplier participates in the negotiations independently and can obtain more profits.

### 7.2 Policy recommendations

Implementing the dual credit policy can stimulate the NEVs supply chain to increase technological innovation and achieve technological breakthroughs. The government can promote the NEVs’ development by guiding or adjusting the credit factor of technical innovation or credit prices.

For manufacturers, NEVs’ technological innovation costs are high, independent technological innovation cost pressure is high risk but may also be limited by technical bottlenecks, and technological innovation cooperation can be a shortcut. Under the dual credit policy, with the increase of the credit factor of technological innovation or the credits price, the technological innovation of the NEVs increase, and the more pronounced the centralization advantage of the supply chain. Therefore, the government can regulate the NEVs industry by setting or adjusting the credit factor of technological innovation or the credit price, guiding the NEVs supply chain to conduct collaborative technological innovation, promoting the healthy and sustainable development of the NEVs industry, and opening a new situation for the development of the NEVs industry.

Furthermore, the government should encourage suppliers to form alliances to provide manufacturers with preinstalled modules, and manufacturers should conduct modular production, which can increase the profit distribution of suppliers within the NEVs supply chain, improve the enthusiasm of suppliers for technological innovation, improve the NEVs technical performance, and then promote the NEVs industry healthy development.

## Supporting information

S1 File (DOCX)

## Abbreviations

NEV: new energy vehicle
Dual credit policy: Regulations on Average Fuel Consumption and New Energy Vehicle Credits of Passenger Vehicle Enterprises
Subscripts *m*: the manufacturer
B: the alliance
*j* = 1,2,…,*n*: the supplier
Superscripts *D*: decentralized decision-making
C: centralized decision-making
Parameters *a*: the potential market demand
θ: the consumer preference coefficient for NEVs technology
p_e_: NEV credits price
σ: NEV credits with the technical performance *T*_0_
I_j_: the technology innovation investment of supplier *j*
T_m_: the technology innovation of manufacturer
π_m_: manufacturer’s profit
B: the supplier alliances
i: the round of nogetiation
β_j_: the supplier’s bargaining power
p: NEVs’ price
w: the parts’ price
λ: technology innovation credits coefficient
k_j_: the technology innovation cost coefficient of supplier *j*
T_j_: the technology innovation of supplier *j*
T_o_: the initial technical level
π_j_: the profit of supplier *j*
ξ: the number of equal-sized supplier alliance
α_j_: the manufacturer’s bargaining power
β_B_: the alliance’s bargaining power
