# Supplementary material for: New energy vehicles’ technology innovation coordination strategy based on alliance negotiation under dual credit policy
Source: PLoS One. 2024 Mar 15;19(3):e0299915. doi: 10.1371/journal.pone.0299915 (PMC10942065; doi:10.1371/journal.pone.0299915)
Supplement: S1 File — (DOCX) [file pone.0299915.s001.docx]

**Appendices**

**Appendix A**

**The optimal strategy-solving process under decentralized decisions**

When the manufacturer and suppliers’ technological innovation investment is a one-time investment, the cost coefficient is usually much more significant than other parameters (Xia et al.,2020).

In decentralized decision-making, let $w_{i}$ and $T_{i}$ differentiate $\pi_{i}$, We can obtain the Hessian matrix of supplier profits is $\left[ \begin{matrix} \frac{\partial^{2}\pi_{j}}{\partial w_{i}^{2}} & \frac{\partial^{2}\pi_{j}}{\partial w_{i}\partial T_{i}} \\ \frac{\partial^{2}\pi_{j}}{\partial T_{i}\partial w_{i}} & \frac{\partial^{2}\pi_{j}}{\partial T_{i}^{2}} \end{matrix} \right]=\left[ \begin{matrix} -2 & \theta\\ \theta& -k_{1} \end{matrix} \right]$.

The first-order principle of the Hessian matrix is $-2<0$

The second-order principle of the Hessian matrix is $\left[ \begin{matrix} -2 & \theta\\ \theta& -k_{1} \end{matrix} \right]=2k_{1}-\theta^{2}>0$.

Therefore, the Hessian matrix of supplier profit is negative definite, $\pi_{i}$ is a concave function of $w_{i}$ and $T_{i}$, and there is a maximum. By simultaneously solving $\frac{\partial\pi_{j}}{\partial w_{j}}=0$ and $\frac{\partial\pi_{j}}{\partial T_{j}}=0$, we can obtain $w_{j}^{D*}=\frac{Z(a-\omega+\theta T_{m})}{(n+1)Z-\theta^{2}M}$,$T_{j}^{D*}=\frac{Z(a-\omega+\theta T_{m})}{(n+1)Z-\theta^{2}M}$ ,where $M=\sum_{\delta=1}^{n} \frac{\prod_{j=1}^{n} k_{j}}{k_{\delta}}k_{m}$, $Z=\prod_{j=1}^{n} k_{j}$. $w_{j}^{D*}$ and $T_{j}^{D*}$ are the unique equilibrium solutions when the supplier's profit maximizes.

In decentralized decision-making, substituting $w_{j}^{D*}$,$T_{j}^{D*}$ into Equation (2), let $\omega$ and $T_{m}$ differentiate $\pi_{m}$, We can obtain the Hessian matrix of manufacturer profits is $\left[ \begin{matrix} \frac{\partial^{2}\pi_{m}}{\partial\omega^{2}} & \frac{\partial^{2}\pi_{m}}{\partial\omega\partial T_{m}} \\ \frac{\partial^{2}\pi_{m}}{\partial T_{m}\partial\omega} & \frac{\partial^{2}\pi_{i}}{\partial\omega^{2}} \end{matrix} \right]=\left[ \begin{matrix} -2 & \theta-p_{e}\lambda\\ \theta-p_{e}\lambda& -k_{m}+2\theta p_{e}\lambda\end{matrix} \right]$.

The first-order principal of the Hessian matrix is $-2<0$

The second-order principal of the Hessian matrix is $\left[ \begin{matrix} -2 & \theta-p_{e}\lambda\\ \theta-p_{e}\lambda& -k_{m}+2\theta p_{e}\lambda\end{matrix} \right]=2(k_{m}-2\theta p_{e}\lambda)-{(\theta-p_{e}\lambda)}^{2}=2k_{m}-{(\theta+p_{e}\lambda)}^{2}$. According to the assumption that the cost $k_{m}$, $k_{j}$ are usually much more significant than other parameters, we can get $\left[ \begin{matrix} -2 & \theta-p_{e}\lambda\\ \theta-p_{e}\lambda& -k_{m}+2\theta p_{e}\lambda\end{matrix} \right]=2k_{m}-{(\theta+p_{e}\lambda)}^{2}>0$.

Therefore, the Hessian matrix of manufacturer profit is negative definite, $\pi_{m}$ is a concave function of $\omega$ and $T_{m}$, and there is a maximum.

By simultaneously solving $\frac{\partial\pi_{m}}{\partial\omega}=0$ and $\frac{\partial\pi_{m}}{\partial T_{m}}=0$, we can obtain $\omega^{D*}=\frac{a\left( \left( n+1 \right)k_{m}Z-p_{e}\lambda\left( \theta+p_{e}\lambda\right)Z-\theta\left( \theta+2p_{e}\lambda\right)M \right)}{Z\left( 2nk_{m}+N \right)-2\theta\left( \theta+p_{e}\lambda\right)M}$,$T_{m}^{D*}=\frac{aZ\left( \theta+p_{e}\lambda\right)}{Z\left( 2nk_{m}+N \right)-2\theta\left( \theta+p_{e}\lambda\right)M}$ ,$p^{D*}=\omega^{D*}+\sum_{i=1}^{n} w_{i}^{D*}$,where $N=2k_{m}-{(\theta+p_{e}\lambda)}^{2}$. $\omega^{D*}$ and $T_{m}^{D*}$ are the unique equilibrium solutions when the manufacturer’s profit maximizes.

Q.E.D.

**Appendix B**

**The optimal strategy-solving process under centralized decisions**

As the technological innovation investment is a one-time investment, the cost is usually much more significant than other parameters.

In decentralized decision-making, let $\pi^{C}$ be differentiated by $T_{m}$ , $T_{i}$ and $p$, We can obtain the Hessian matrix of NEV supply chai profits is $\left[ \begin{matrix} \begin{matrix} \frac{\partial^{2}\pi^{C}}{\partial p^{2}} & \frac{\partial^{2}\pi^{C}}{\partial p\partial T_{m}} & \begin{matrix} \frac{\partial^{2}\pi^{C}}{\partial p\partial T_{1}} & \cdots\end{matrix} \end{matrix} & \frac{\partial^{2}\pi^{C}}{\partial p\partial T_{n}} \\ \begin{matrix} \begin{matrix} \frac{\partial^{2}\pi^{C}}{\partial T_{m}\partial p} \\ \begin{matrix} \frac{\partial^{2}\pi^{C}}{\partial T_{1}\partial p} \\ \cdots\\ \frac{\partial^{2}\pi^{C}}{\partial T_{n}\partial p} \end{matrix} \end{matrix} & \begin{matrix} \frac{\partial^{2}\pi^{C}}{\partial T_{m}^{2}} \\ \begin{matrix} \frac{\partial^{2}\pi^{C}}{\partial T_{1}\partial T_{m}} \\ \cdots\\ \frac{\partial^{2}\pi^{C}}{\partial T_{n}\partial T_{m}} \end{matrix} \end{matrix} & \begin{matrix} \begin{matrix} \frac{\partial^{2}\pi^{C}}{\partial T_{m}\partial T_{1}} & \cdots\end{matrix} \\ \begin{matrix} \begin{matrix} \frac{\partial^{2}\pi^{C}}{\partial T_{1}^{2}} & \cdots\end{matrix} \\ \begin{matrix} \cdots& \cdots\end{matrix} \\ \begin{matrix} \frac{\partial^{2}\pi^{C}}{\partial T_{n}\partial T_{1}} & \cdots\end{matrix} \end{matrix} \end{matrix} \end{matrix} & \begin{matrix} \frac{\partial^{2}\pi^{C}}{\partial T_{1}\partial T_{n}} \\ \begin{matrix} \frac{\partial^{2}\pi^{C}}{\partial T_{1}\partial T_{n}} \\ \cdots\\ \frac{\partial^{2}\pi^{C}}{\partial T_{n}^{2}} \end{matrix} \end{matrix} \end{matrix} \right]=\left[ \begin{matrix} \begin{matrix} -2 & \theta-p_{e}\lambda& \cdots\end{matrix} & \begin{matrix} \theta-p_{e}\lambda& \theta-p_{e}\lambda\end{matrix} \\ \begin{matrix} \begin{matrix} \theta-p_{e}\lambda& 2\theta p_{e}\lambda-k_{m} & \cdots\end{matrix} \\ \begin{matrix} \theta-p_{e}\lambda& 2\theta p_{e}\lambda& 2\theta p_{e}\lambda\end{matrix}-k_{1} \\ \begin{matrix} \begin{matrix} \cdots\\ \theta-p_{e}\lambda\end{matrix} & \begin{matrix} \cdots\\ 2\theta p_{e}\lambda\end{matrix} & \begin{matrix} \cdots\\ 2\theta p_{e}\lambda\end{matrix} \end{matrix} \end{matrix} & \begin{matrix} \begin{matrix} 2\theta p_{e}\lambda& 2\theta p_{e}\lambda\end{matrix} \\ \begin{matrix} \cdots& 2\theta p_{e}\lambda\end{matrix} \\ \begin{matrix} \cdots& \begin{matrix} \cdots\\ 2\theta p_{e}\lambda-k_{n} \end{matrix} \end{matrix} \end{matrix} \end{matrix} \right]$

The first-order principle of the Hessian matrix is $-2<0$.

The second-order principle of the Hessian matrix is $\left[ \begin{matrix} -2 & \theta-p_{e}\lambda\\ \theta-p_{e}\lambda& 2\theta p_{e}\lambda-k_{m} \end{matrix} \right]=2(k_{m}-2\theta p_{e}\lambda)-{(\theta+p_{e}\lambda)}^{2}=2k_{m}-{(\theta+p_{e}\lambda)}^{2}$. According to the assumption that the cost $k_{m}$, $k_{j}$ are usually much more significant than other parameters, we can get $\left[ \begin{matrix} -2 & \theta-p_{e}\lambda\\ \theta-p_{e}\lambda& 2\theta p_{e}\lambda-k_{m} \end{matrix} \right]=2k_{m}-\left( \theta+p_{e}\lambda\right)^{2}>0$.

The third-order principal of the Hessian matrix is $\left| \begin{matrix} \begin{matrix} -2 & \theta-p_{e}\lambda\end{matrix} & \theta-p_{e}\lambda\\ \begin{matrix} \begin{matrix} \theta-p_{e}\lambda& 2\theta p_{e}\lambda-k_{m} \end{matrix} \\ \begin{matrix} \theta-p_{e}\lambda& 2\theta p_{e}\lambda\end{matrix} \end{matrix} & \begin{matrix} 2\theta p_{e}\lambda\\ 2\theta p_{e}\lambda-k_{1} \end{matrix} \end{matrix} \right|=-2\left( 2\theta p_{e}\lambda-k_{m} \right)\left( 2\theta p_{e}\lambda-k_{1} \right)+{4\theta p_{e}\lambda\left( \theta-p_{e}\lambda\right)}^{2}-{\left( 2\theta p_{e}\lambda-k_{m} \right)\left( \theta-p_{e}\lambda\right)}^{2}-{\left( 2\theta p_{e}\lambda-k_{1} \right)\left( \theta-p_{e}\lambda\right)}^{2}+8\theta^{2}{{p_{e}}^{2}\lambda}^{2}=-2k_{1}k_{m}+\left( k_{1}+k_{m} \right)\left( \theta+p_{e}\lambda\right)^{2}=-{(k}_{1}\left( k_{m}-\left( \theta+p_{e}\lambda\right)^{2} \right)+k_{m}(k_{1}-\left( \theta+p_{e}\lambda\right)^{2}))$. According to the assumption that the cost $k_{m}$, $k_{j}$ are usually much more significant than other parameters, we can get $\left| \begin{matrix} \begin{matrix} -2 & \theta-p_{e}\lambda\end{matrix} & \theta-p_{e}\lambda\\ \begin{matrix} \begin{matrix} \theta-p_{e}\lambda& 2\theta p_{e}\lambda-k_{m} \end{matrix} \\ \begin{matrix} \theta-p_{e}\lambda& 2\theta p_{e}\lambda\end{matrix} \end{matrix} & \begin{matrix} 2\theta p_{e}\lambda\\ 2\theta p_{e}\lambda-k_{1} \end{matrix} \end{matrix} \right|=-2k_{1}k_{m}+\left( k_{1}+k_{m} \right)\left( \theta+p_{e}\lambda\right)^{2}<0$.

The fourth-order principle of the Hessian matrix is $\left| \begin{matrix} \begin{matrix} -2 & \theta-p_{e}\lambda& \theta-p_{e}\lambda\end{matrix} & \theta-p_{e}\lambda\\ \begin{matrix} \begin{matrix} \theta-p_{e}\lambda\\ \theta-p_{e}\lambda\\ \theta-p_{e}\lambda\end{matrix} & \begin{matrix} 2\theta p_{e}\lambda-k_{m} \\ \theta-p_{e}\lambda\\ 2\theta p_{e}\lambda\end{matrix} & \begin{matrix} 2\theta p_{e}\lambda\\ 2\theta p_{e}\lambda-k_{1} \\ 2\theta p_{e}\lambda\end{matrix} \end{matrix} & \begin{matrix} 2\theta p_{e}\lambda\\ 2\theta p_{e}\lambda\\ 2\theta p_{e}\lambda-k_{2} \end{matrix} \end{matrix} \right|=-2(2\theta\lambda-k_{m})(2\theta\lambda-k_{1})(2\theta p_{e}\lambda-k_{2})+{8{\theta^{2}{p_{e}}^{2}\lambda}^{2}(\theta-p_{e}\lambda)}^{2}+2\theta p_{e}\lambda{(\theta-p_{e}\lambda)}^{3}-2\theta p_{e}\lambda{(\theta-p_{e}\lambda)}^{3}{-(2\theta p_{e}\lambda-k_{m})(2\theta p_{e}\lambda-k_{2})(\theta-p_{e}\lambda)}^{2}+8{\theta^{2}{p_{e}}^{2}\lambda}^{2}(2\theta p_{e}\lambda-k_{1})-4\theta^{2}{{p_{e}}^{2}\lambda}^{2}{(\theta-p_{e}\lambda)}^{2}=2k_{1}k_{2}k_{m}-(k_{1}k_{2}+k_{1}k_{m}+k_{2}k_{m}){(\theta+p_{e}\lambda)}^{2}$.According to the assumption that $\prod_{j=1}^{n} k_{j}-\sum_{\delta=1}^{n} \frac{\prod_{j=1}^{n} k_{j}}{k_{\delta}}k_{m}{(\theta+p_{e}\lambda)}^{2}>0$, we can get $\left| \begin{matrix} \begin{matrix} -2 & \theta-p_{e}\lambda& \theta-p_{e}\lambda\end{matrix} & \theta-p_{e}\lambda\\ \begin{matrix} \begin{matrix} \theta-p_{e}\lambda\\ \theta-p_{e}\lambda\\ \theta-p_{e}\lambda\end{matrix} & \begin{matrix} 2\theta p_{e}\lambda-k_{m} \\ \theta-p_{e}\lambda\\ 2\theta p_{e}\lambda\end{matrix} & \begin{matrix} 2\theta p_{e}\lambda\\ 2\theta p_{e}\lambda-k_{1} \\ 2\theta p_{e}\lambda\end{matrix} \end{matrix} & \begin{matrix} 2\theta p_{e}\lambda\\ 2\theta p_{e}\lambda\\ 2\theta p_{e}\lambda-k_{2} \end{matrix} \end{matrix} \right|=2k_{1}k_{2}k_{m}-(k_{1}k_{2}+k_{1}k_{m}+k_{2}k_{m}){(\theta+p_{e}\lambda)}^{2}>0$.

And so on, the odd-degree order principal of matrix is less than 0, and the even-degree order principal is greater than 0.

Therefore, the Hessian matrix of NEV supply chain profit is negative definite, $\pi^{c}$ is a concave function of $p$, $T$ and $T_{m}$, and there is a maximum.

By simultaneously solving $\frac{\partial\pi^{C}}{\partial T_{m}}=0$, $\frac{\partial\pi^{C}}{\partial T_{j}}=0$ and $\frac{\partial\pi_{m}}{\partial T_{m}}=0$, we can obtain $T_{j}^{C*}=\frac{{ak}_{m}Z(\theta+p_{e}\lambda)}{k_{j}(ZN-{(\theta+p_{e}\lambda)}^{2}M)}$ , $T_{m}^{C*}=\frac{aZ(\theta+p_{e}\lambda)}{ZN-{(\theta+p_{e}\lambda)}^{2}M}$ , $p^{C*}=\frac{a(k_{m}N-\lambda(\theta+p_{e}\lambda)M)}{ZN-M{(\theta+p_{e}\lambda)}^{2}}$. $T_{j}^{C*}$, $T_{m}^{C*}$and $p^{C*}$ are the unique equilibrium solutions when the supply chain’s profit maximizes.

Q.E.D.

**Appendix C**

**The proof of Proposition 1.**

*Proof.* let $\boldsymbol{I}_{\boldsymbol{j}}^{\boldsymbol{D*}}$, $\boldsymbol{I}_{\boldsymbol{m}}^{\boldsymbol{D*}}$, $\boldsymbol{I}_{\boldsymbol{j}}^{\boldsymbol{C*}}$, $\boldsymbol{I}_{\boldsymbol{m}}^{\boldsymbol{C*}}$, $\boldsymbol{q}^{\boldsymbol{D*}}$, $\boldsymbol{q}^{\boldsymbol{C*}}$, $\boldsymbol{T}_{\boldsymbol{j}}^{\boldsymbol{D*}}$, $\boldsymbol{T}_{\boldsymbol{m}}^{\boldsymbol{D*}}$, $\boldsymbol{T}_{\boldsymbol{j}}^{\boldsymbol{C*}}$, $\boldsymbol{T}_{\boldsymbol{m}}^{\boldsymbol{C*}}$ , $\boldsymbol{\pi}^{\boldsymbol{C*}}$ , $\boldsymbol{\pi}^{\boldsymbol{D*}}$ be differentiated by $\boldsymbol{\lambda}$ and $\boldsymbol{p}_{\boldsymbol{e}}$, respectively, we can obtain $\frac{\boldsymbol{\partial}\boldsymbol{I}_{\boldsymbol{j}}^{\boldsymbol{D*}}}{\boldsymbol{\partial\lambda}}\boldsymbol{=}\frac{\boldsymbol{a}^{\boldsymbol{2}}\boldsymbol{k}_{\boldsymbol{m}}^{\boldsymbol{2}}\boldsymbol{\theta}^{\boldsymbol{2}}\boldsymbol{Z}^{\boldsymbol{2}}\left( \boldsymbol{2\theta}\boldsymbol{p}_{\boldsymbol{e}}\boldsymbol{M+2Z}\boldsymbol{p}_{\boldsymbol{e}}\left( \boldsymbol{\theta+}\boldsymbol{p}_{\boldsymbol{e}}\boldsymbol{\lambda} \right) \right)}{{\boldsymbol{k}_{\boldsymbol{j}}\left( \left( \left( \boldsymbol{2n+2} \right)\boldsymbol{k}_{\boldsymbol{m}}\boldsymbol{-}\left( \boldsymbol{\theta+}\boldsymbol{p}_{\boldsymbol{e}}\boldsymbol{\lambda} \right)^{\boldsymbol{2}} \right)\boldsymbol{Z-2\theta}\left( \boldsymbol{\theta+}\boldsymbol{p}_{\boldsymbol{e}}\boldsymbol{\lambda} \right)\boldsymbol{M} \right)}^{\boldsymbol{3}}}\boldsymbol{>0}$，$\frac{\boldsymbol{\partial}\boldsymbol{I}_{\boldsymbol{m}}^{\boldsymbol{D*}}}{\boldsymbol{\partial\lambda}}\boldsymbol{=}\frac{\boldsymbol{a}^{\boldsymbol{2}}{\boldsymbol{k}_{\boldsymbol{m}}\boldsymbol{Z}}^{\boldsymbol{3}}\boldsymbol{p}_{\boldsymbol{e}}\left( \left( \boldsymbol{2n+2} \right)\boldsymbol{k}_{\boldsymbol{m}}\boldsymbol{+}\left( \boldsymbol{\theta+}\boldsymbol{p}_{\boldsymbol{e}}\boldsymbol{\lambda} \right)^{\boldsymbol{2}} \right)\left( \boldsymbol{\theta+}\boldsymbol{p}_{\boldsymbol{e}}\boldsymbol{\lambda} \right)}{\left( \left( \boldsymbol{2n}\boldsymbol{k}_{\boldsymbol{m}}\boldsymbol{+N} \right)\boldsymbol{Z-2\theta}\left( \boldsymbol{\theta+}\boldsymbol{p}_{\boldsymbol{e}}\boldsymbol{\lambda} \right)\boldsymbol{M} \right)^{\boldsymbol{3}}}\boldsymbol{>0}$，$\frac{\boldsymbol{\partial}\boldsymbol{T}_{\boldsymbol{j}}^{\boldsymbol{D*}}}{\boldsymbol{\partial\lambda}}\boldsymbol{=}\frac{\boldsymbol{2a}\boldsymbol{k}_{\boldsymbol{m}}\boldsymbol{Z\theta}\left( \boldsymbol{2}\boldsymbol{p}_{\boldsymbol{e}}\boldsymbol{\theta M+2}\boldsymbol{p}_{\boldsymbol{e}}\boldsymbol{Z}\left( \boldsymbol{\theta+}\boldsymbol{p}_{\boldsymbol{e}}\boldsymbol{\lambda} \right) \right)}{{\boldsymbol{k}_{\boldsymbol{i}}\left( \left( \boldsymbol{2n}\boldsymbol{k}_{\boldsymbol{m}}\boldsymbol{+N} \right)\boldsymbol{Z-2\theta}\left( \boldsymbol{\theta+}\boldsymbol{p}_{\boldsymbol{e}}\boldsymbol{\lambda} \right)\boldsymbol{M} \right)}^{\boldsymbol{2}}}\boldsymbol{>0}$，$\frac{\boldsymbol{\partial}\boldsymbol{T}_{\boldsymbol{m}}^{\boldsymbol{D*}}}{\boldsymbol{\partial\lambda}}\boldsymbol{=}\frac{\boldsymbol{a}\boldsymbol{p}_{\boldsymbol{e}}\boldsymbol{Z}^{\boldsymbol{2}}\left( \left( \boldsymbol{2n+2} \right)\boldsymbol{k}_{\boldsymbol{m}}\boldsymbol{+}\left( \boldsymbol{\theta+}\boldsymbol{p}_{\boldsymbol{e}}\boldsymbol{\lambda} \right)^{\boldsymbol{2}} \right)}{\left( \left( \boldsymbol{2n}\boldsymbol{k}_{\boldsymbol{m}}\boldsymbol{+N} \right)\boldsymbol{Z-2\theta}\left( \boldsymbol{\theta+}\boldsymbol{p}_{\boldsymbol{e}}\boldsymbol{\lambda} \right)\boldsymbol{M} \right)^{\boldsymbol{2}}}\boldsymbol{>0}$，$\frac{\boldsymbol{\partial}\boldsymbol{q}^{\boldsymbol{D*}}}{\boldsymbol{\partial\lambda}}\boldsymbol{=}\frac{\boldsymbol{2a}\boldsymbol{k}_{\boldsymbol{m}}\boldsymbol{Z}\left( \boldsymbol{M\theta}\boldsymbol{p}_{\boldsymbol{e}}\boldsymbol{+}\boldsymbol{p}_{\boldsymbol{e}}\boldsymbol{Z}\left( \boldsymbol{\theta+}\boldsymbol{p}_{\boldsymbol{e}}\boldsymbol{\lambda} \right) \right)}{\left( \left( \boldsymbol{2n}\boldsymbol{k}_{\boldsymbol{m}}\boldsymbol{+N} \right)\boldsymbol{Z-2\theta}\left( \boldsymbol{\theta+}\boldsymbol{p}_{\boldsymbol{e}}\boldsymbol{\lambda} \right)\boldsymbol{M} \right)^{\boldsymbol{2}}}\boldsymbol{>0}$，$\frac{\boldsymbol{\partial}\boldsymbol{\pi}^{\boldsymbol{D}}}{\boldsymbol{\partial\lambda}}\boldsymbol{=}\frac{\boldsymbol{a}^{\boldsymbol{2}}\boldsymbol{k}_{\boldsymbol{m}}\boldsymbol{Z}\boldsymbol{p}_{\boldsymbol{e}}\boldsymbol{(\theta M+Z(\theta+}\boldsymbol{p}_{\boldsymbol{e}}\boldsymbol{\lambda))(2(1+3n)}\boldsymbol{k}_{\boldsymbol{m}}\boldsymbol{-}\left( \boldsymbol{\theta+}\boldsymbol{p}_{\boldsymbol{e}}\boldsymbol{\lambda} \right)^{\boldsymbol{2}}\boldsymbol{)Z-2M\theta(2\theta+}\boldsymbol{p}_{\boldsymbol{e}}\boldsymbol{\lambda))}}{\left( \left( \left( \boldsymbol{2n+2} \right)\boldsymbol{k}_{\boldsymbol{m}}\boldsymbol{-}\left( \boldsymbol{\theta+}\boldsymbol{p}_{\boldsymbol{e}}\boldsymbol{\lambda} \right)^{\boldsymbol{2}} \right)\boldsymbol{Z-2\theta}\left( \boldsymbol{\theta+}\boldsymbol{p}_{\boldsymbol{e}}\boldsymbol{\lambda} \right)\boldsymbol{M} \right)^{\boldsymbol{3}}}\boldsymbol{>0}$，$\frac{\boldsymbol{\partial}\boldsymbol{I}_{\boldsymbol{j}}^{\boldsymbol{C*}}}{\boldsymbol{\partial\lambda}}\boldsymbol{=}\frac{\boldsymbol{p}_{\boldsymbol{e}}{\boldsymbol{(a}\boldsymbol{k}_{\boldsymbol{m}}\boldsymbol{Z)}}^{\boldsymbol{2}}\boldsymbol{(\theta+}\boldsymbol{p}_{\boldsymbol{e}}\boldsymbol{\lambda)(2}\boldsymbol{k}_{\boldsymbol{m}}\boldsymbol{Z+(M+Z)}{\boldsymbol{(\theta+}\boldsymbol{p}_{\boldsymbol{e}}\boldsymbol{\lambda)}}^{\boldsymbol{2}}\boldsymbol{)}}{{\boldsymbol{(NZ-M}{\boldsymbol{(\theta+}\boldsymbol{p}_{\boldsymbol{e}}\boldsymbol{\lambda)}}^{\boldsymbol{2}}\boldsymbol{)}}^{\boldsymbol{3}}}\boldsymbol{>0}$,$\frac{\boldsymbol{\partial}\boldsymbol{I}_{\boldsymbol{m}}^{\boldsymbol{C*}}}{\boldsymbol{\partial\lambda}}\boldsymbol{=}\frac{\boldsymbol{p}_{\boldsymbol{e}}\boldsymbol{k}_{\boldsymbol{m}}\boldsymbol{(aZ)}^{\boldsymbol{2}}\boldsymbol{(\theta+}\boldsymbol{p}_{\boldsymbol{e}}\boldsymbol{\lambda)(2}\boldsymbol{k}_{\boldsymbol{m}}\boldsymbol{Z+(M+Z)}{\boldsymbol{(\theta+}\boldsymbol{p}_{\boldsymbol{e}}\boldsymbol{\lambda)}}^{\boldsymbol{2}}\boldsymbol{)}}{{\boldsymbol{(NZ-M}{\boldsymbol{(\theta+}\boldsymbol{p}_{\boldsymbol{e}}\boldsymbol{\lambda)}}^{\boldsymbol{2}}\boldsymbol{)}}^{\boldsymbol{3}}}$,$\frac{\boldsymbol{\partial}\boldsymbol{T}_{\boldsymbol{j}}^{\boldsymbol{C*}}}{\boldsymbol{\partial\lambda}}\boldsymbol{=}\frac{\boldsymbol{aZp}_{\boldsymbol{e}}\boldsymbol{k}_{\boldsymbol{m}}\boldsymbol{(2}\boldsymbol{k}_{\boldsymbol{m}}\boldsymbol{Z+(M+Z)}{\boldsymbol{(\theta+}\boldsymbol{p}_{\boldsymbol{e}}\boldsymbol{\lambda)}}^{\boldsymbol{2}}\boldsymbol{)}}{\boldsymbol{k}_{\boldsymbol{i}}{\boldsymbol{(NZ-M}{\boldsymbol{(\theta+}\boldsymbol{p}_{\boldsymbol{e}}\boldsymbol{\lambda)}}^{\boldsymbol{2}}\boldsymbol{)}}^{\boldsymbol{2}}}\boldsymbol{>0}$,$\frac{\boldsymbol{\partial}\boldsymbol{T}_{\boldsymbol{m}}^{\boldsymbol{C*}}}{\boldsymbol{\partial\lambda}}\boldsymbol{=}\frac{\boldsymbol{aZp}_{\boldsymbol{e}}\boldsymbol{(2}\boldsymbol{k}_{\boldsymbol{m}}\boldsymbol{Z+(M+Z)}{\boldsymbol{(\theta+}\boldsymbol{p}_{\boldsymbol{e}}\boldsymbol{\lambda)}}^{\boldsymbol{2}}\boldsymbol{)}}{{\boldsymbol{(NZ-M}{\boldsymbol{(\theta+}\boldsymbol{p}_{\boldsymbol{e}}\boldsymbol{\lambda)}}^{\boldsymbol{2}}\boldsymbol{)}}^{\boldsymbol{2}}}\boldsymbol{>0}$,$\frac{\boldsymbol{\partial}\boldsymbol{q}^{\boldsymbol{C*}}}{\boldsymbol{\partial\lambda}}\boldsymbol{=}\frac{\boldsymbol{2aZ}\boldsymbol{k}_{\boldsymbol{m}}\boldsymbol{p}_{\boldsymbol{e}}\boldsymbol{(\theta+}\boldsymbol{p}_{\boldsymbol{e}}\boldsymbol{\lambda)(M+Z)}}{{\boldsymbol{(NZ-M}{\boldsymbol{(\theta+}\boldsymbol{p}_{\boldsymbol{e}}\boldsymbol{\lambda)}}^{\boldsymbol{2}}\boldsymbol{)}}^{\boldsymbol{2}}}\boldsymbol{>0}$, $\frac{\boldsymbol{\partial}\boldsymbol{\pi}^{\boldsymbol{C}}}{\boldsymbol{\partial\lambda}}\boldsymbol{=}\frac{\boldsymbol{2}\boldsymbol{a}^{\boldsymbol{2}}\boldsymbol{p}_{\boldsymbol{e}}\boldsymbol{k}_{\boldsymbol{m}}\boldsymbol{Z(M+Z)(\theta+}\boldsymbol{p}_{\boldsymbol{e}}\boldsymbol{\lambda)}}{{\boldsymbol{(NZ-M}{\boldsymbol{(\theta+}\boldsymbol{p}_{\boldsymbol{e}}\boldsymbol{\lambda)}}^{\boldsymbol{2}}\boldsymbol{)}}^{\boldsymbol{3}}}\boldsymbol{>0}$*.*

By the same token, we can get $\frac{\partial I_{j}^{D*}}{\partial p_{e}}>0$, $\frac{\partial I_{m}^{D*}}{\partial p_{e}}>0$,$\frac{\partial T_{j}^{D*}}{\partial p_{e}}>0$, $\frac{\partial T_{m}^{D*}}{\partial p_{e}}>0$, $\frac{\partial q^{D*}}{\partial p_{e}}>0$, $\frac{\partial\pi^{D}}{\partial p_{e}}>0$, $\frac{\partial I_{j}^{C*}}{\partial p_{e}}>0$, $\frac{\partial I_{m}^{C*}}{\partial p_{e}}>0$, $\frac{\partial T_{j}^{C*}}{\partial p_{e}}>0$, $\frac{\partial q^{C*}}{\partial p_{e}}>0$, $\frac{\partial\pi^{C}}{\partial p_{e}}>0$.

Q.E.D.

**Appendix D**

**The proof of Proposition 2.**

*Proof.* Substituting $T_{m}^{D*}$, $T_{j}^{D*}$, $T_{j}^{C*}$, $T_{m}^{C*}$, $\pi^{C*}$, and $\pi^{D*}$ into $T_{m}^{C*}-T_{m}^{D*}$,

$T_{j}^{C*}-T_{j}^{D*}$, and $\pi^{C*}-\pi^{D*}$, respectively, we can obtain

$T_{m}^{C*}-T_{m}^{D*}=\frac{aZ(\theta+p_{e}\lambda)(2{nk}_{m}Z-(({p_{e}\lambda)}^{2}+\theta^{2})M)}{(ZN-{(\theta+p_{e}\lambda)}^{2}M)(Z(2nk_{m}+N)-2\theta(\theta+p_{e}\lambda)M)}>0$,

$T_{j}^{C*}-T_{j}^{D*}=\frac{{ak}_{m}Z(\theta((2{nk}_{m}Z-\theta^{2}M)+p_{e}\lambda(c(2nk_{m}+N)-2\theta(\theta+p_{e}\lambda)M))}{k_{j}(ZN-{(\theta+p_{e}\lambda)}^{2}M)(Z(2nk_{m}+N)-2\theta(\theta+p_{e}\lambda)M))}>0$,

$\pi^{C*}-\pi^{D*}=\frac{a^{2}Zk_{m}(4nk_{m}Z(nZ-\theta^{2}M)+({p_{e}\lambda)}^{2}M(Z(4nk_{m}+N)-\theta(3\theta+2p_{e}\lambda)+\theta^{4}M^{2})}{2{(Z(2nk_{m}+N)-2\theta(\theta+p_{e}\lambda)M)}^{2}(ZN-{(\theta+p_{e}\lambda)}^{2}M)}>0$

Q.E.D.
